# Construction of an annotated corpus to support biomedical information extraction

**DOI:** 10.1186/1471-2105-10-349

**Published:** 2009-10-23

**Authors:** Paul Thompson, Syed A Iqbal, John McNaught, Sophia Ananiadou

**Affiliations:** 1 National Centre for Text Mining, Manchester Interdisciplinary Biocentre, University of Manchester, 131 Princess Street, Manchester, M1 7DN, UK

## Abstract

**Background:**

Information Extraction (IE) is a component of text mining that facilitates knowledge discovery by automatically locating instances of interesting biomedical events from huge document collections. As events are usually centred on verbs and nominalised verbs, understanding the syntactic and semantic behaviour of these words is highly important. Corpora annotated with information concerning this behaviour can constitute a valuable resource in the training of IE components and resources.

**Results:**

We have defined a new scheme for annotating sentence-bound gene regulation events, centred on both verbs and nominalised verbs. For each event instance, all participants (*arguments*) in the same sentence are identified and assigned a semantic role from a rich set of 13 roles tailored to biomedical research articles, together with a biological concept type linked to the Gene Regulation Ontology. To our knowledge, our scheme is unique within the biomedical field in terms of the range of event arguments identified. Using the scheme, we have created the Gene Regulation Event Corpus (GREC), consisting of 240 MEDLINE abstracts, in which events relating to gene regulation and expression have been annotated by biologists. A novel method of evaluating various different facets of the annotation task showed that average inter-annotator agreement rates fall within the range of 66% - 90%.

**Conclusion:**

The GREC is a unique resource within the biomedical field, in that it annotates not only core relationships between entities, but also a range of other important details about these relationships, e.g., location, temporal, manner and environmental conditions. As such, it is specifically designed to support bio-specific tool and resource development. It has already been used to acquire semantic frames for inclusion within the *BioLexicon *(a lexical, terminological resource to aid biomedical text mining). Initial experiments have also shown that the corpus may viably be used to train IE components, such as semantic role labellers. The corpus and annotation guidelines are freely available for academic purposes.

## Background

Due to the rapid advances in biomedical research, scientific literature is being published at an ever-increasing rate [1]. Without automated means, it is difficult for researchers to keep abreast of developments within biomedicine [2-6]. Text mining, which is receiving increasing interest within the biomedical field [7,8], enriches text via the addition of semantic metadata, and thus permits tasks such as analysing molecular pathways [9] and semantic searching.

Semantic searching above the level of concepts depends on prior processing to recognise relations or *events *in texts, which is carried out by information extraction (IE) systems. Due to domain-specific features of texts and the types of events to be recognised, IE systems must be adapted to deal with specific domains. A well-established method of carrying out this adaptation is through training using annotated corpora (e.g., [10-12]).

Our work has been concerned with the development of such a corpus for the biomedical field, the Gene Regulation Event Corpus (GREC), consisting of MEDLINE abstracts semantically annotated with event information. Our approach is based on the fact that many events are focussed on either verbs (e.g., *transcribe*, *regulate*) or nominalised verbs (e.g., *transcription*, *regulation*). Both types of word behave in similar ways, in that they specify arguments that can convey a range of different types of information related to the event. For each relevant event, our annotation aims to identify, as exhaustively as possible, all structurally-related arguments within the same sentence. Each argument is assigned a semantic role from a fixed set of 13 roles. Where appropriate, arguments are also assigned a biological concept type. The GREC may be downloaded from . A copy of the corpus is also available in Additional file 1.

To our knowledge, the GREC provides the richest annotation to date within the biomedical field, in terms of the number of arguments types and their characterisation. As such, the corpus is specifically designed to contribute to the development of bio-specific semantic frame resources and semantic role labellers (SRLs) which, although active areas of research within the general language domain, have received less attention within the bio-IE domain.

### Related work

Within the field of bio-IE, evaluations such as the LLL05 challenge [13] and BioCreative II [14] have focussed attention on the recognition of protein-protein interaction (PPI) events from the literature. There now exists a number of corpora (e.g., [13,15,16]) and systems (e.g., [17-19]) tailored to this task. However, many other types of events and information are relevant within biomedicine, such as gene regulation and expression events, location of protein in the cell, protein-DNA interaction, etc. [20]. Extraction of such events often requires the recognition of more complex information than just interacting proteins.

Several corpora and systems concerned with the annotation of more complex events have recently been developed, e.g., [21-24]. These differ in a number of ways, including:

• **Range of events **- whether a single type of event or multiple event types are annotated.

• **Event arguments **- the number and types of arguments (i.e., participants) in each event may be fixed or flexible. More detailed information types, e.g., location, time or experimental setup may or may not be identified as event arguments.

• **Scope of events **- whether event arguments must occur within a single sentence or whether they may occur across multiple sentences.

• **Semantic information assigned to arguments **- this may correspond to named entity types and/or semantic roles. In the case of semantic roles being assigned, they may be tailored to a particular type of event, or they may apply to a large range of different events.

Whilst events are often identified by verbs, nominalised verbs play a particularly important role within biomedical texts, and often outnumber other domain-specific verbal forms [25]. However, it is acknowledged that they are more difficult to process than verbs [26] and are currently only dealt with by a small number of systems, often in a limited way (e.g., [17,27,28]).

Due to the central nature of verbs and nominalised verbs in the description of events, accurate event extraction requires information about the way they behave in text, in terms of:

• Their syntactically-related arguments, e.g. causality, location, manner, etc.

• Semantic information relating to each argument (e.g., semantic roles or restrictions on the types of phrase that can constitute each argument).

The production of corpora annotated with such information allows real usage within text to be taken into account. Large-scale annotation of corpora within the general language domain at this level of detail has resulted in the production of resources containing syntactic and semantic frame information, which deal with both verbs and nominalised verbs [29-32]. Such annotated corpora also facilitate the training of components of IE systems, with a large amount of research having been devoted to semantic role labelling (SRL) [33].

Some studies (e.g., [34,35]) have shown that, to a certain extent, general language resources can also be useful in the training of SRLs for biomedical texts, due to the fact that many verbs appear in texts from both the general language and biomedical domains, and often behave in similar ways. However, the cited works concede that, whilst such SRLs may produce adequate results for certain predicates, training using biomedical corpora is also needed. This is because domains such as biomedicine employ sublanguages [36], in which the "informational content and structure form a specialized language that can be delineated in the form of a sublanguage grammar". NLP systems must take such grammars into account to allow accurate processing of text within specialist domains [37]. Sublanguage grammar features that are relevant to our work include the following:

• The types of events found in biological sciences are often described using verbs/nominalised verbs that do not feature prominently in general language [26], e.g., *methylate*.

• Verbs/nominalised verbs that occur in both the general and specialised language domains may have different syntactic and semantic properties in each domain, e.g., differing numbers of arguments [38], as well as different meanings. For example, *translation *generally means rendering one language into other, while in Molecular Biology it specifies the process of protein synthesis from an mRNA template.

Whilst there have been some attempts to produce bio-specific extensions to the general language resources described above, e.g., [38,39], together with semantic role labellers [20,40], they currently have limited coverage. The UMLS SPECIALIST lexicon [41], which includes many biomedical terms, is larger scale, but includes only syntactic, and not semantic, information about verbs.

### Motivation

Existing event corpora within the domain (e.g., [21] and [23]) are not specifically geared to support the acquisition of semantic frame information for verbs. The bio-NLP community has, until now, lacked a domain-specific linguistically-oriented corpus in which detailed semantic information for a wide range of both verbs and nominalised verbs has been annotated. This has limited the amount of research undertaken on the production of domain-specific semantic frame resources and SRLs.

In response to this, we have designed a new event annotation scheme which is specifically tailored to this purpose. The scheme has subsequently been applied to the annotation of event instances relating to gene regulation and expression in MEDLINE abstracts. Our scheme differs from those of previous event corpora in the field in a number of important ways:

• It captures the semantic annotation of as many structurally-related arguments as possible of a large number of verbs and nominalised verbs describing gene regulation and expression events. This is important since, according to [20], and as confirmed by us through consultation with biologists, types of information such as location, manner, timing and condition, which can appear in various syntactic positions, are all essential for describing biomedical relations. A sentence-based approach facilitates the linking between semantic information and syntactic structure.

• It bridges linguistic and biological knowledge:

◦ From the linguistic perspective, all arguments are characterised using semantic roles. We have defined a new, closed set of event-independent roles which are designed for application to arguments of a range of types of biomedical events. Closed sets of semantic roles are advantageous in that they facilitate generalization over different types of events [25,42]. Although their application to general language may be problematic [30], the use of a closed set is viable in a restricted domain, as domain-specific definitions can be provided for each semantic role type.

◦ From the biological perspective, appropriate arguments are additionally assigned a biological concept type from a hierarchically-structured set that is tailored to the gene regulation domain. The concepts are mapped to classes in the Gene Regulation Ontology (GRO) [43].

The combination of semantic role and biological concept labels provides a rich annotation, aimed at allowing users to have a large amount of flexibility over the type of query they specify and to have control over the specificity or generality of certain parts of the query, e.g.:

*In LOCATION:E. coli, AGENT:NifA activates which THEME:GENE*.

This query would search for instances of events in which a LOCATION, AGENT and THEME are specified. The values of the semantic roles may be specified either as specific words or phrases (e.g., *E. coli *or *NifA*) or more general named entity categories (e.g., *GENE*).

The GREC consists of 240 MEDLINE abstracts, which have been annotated with a total of 3067 events. Whilst of modest size compared to some other domain-specific event-annotated corpora (e.g., [21]), this is balanced by the richness of the annotations.

The corpus has already been used in the development of the *BioLexicon *[44]. This unique text mining resource for biology provides and links syntactic and semantic frame information for a large number of biomedical sublanguage verbs. In addition, the lexicon contains (1) derived forms of these verbs (including nominalisations), (2) general English words frequently used within the biology domain and (3) domain terms, gathered (and interlinked) both from existing databases and through the application of text mining techniques.

Initial machine learning experiments using the GREC [45] suggest that it can be used to train IE components with reasonably good performance, with both named entity extraction and semantic role labelling having achieved F-scores of around 60%, based on 10-fold cross validation.

A further direction of research which could help to improve the performance of IE systems trained on the GREC is introduced in [46], in which it is demonstrated that, due to the differing perspectives of different annotation schemes, it is not always the case that larger corpora contain the most useful information. The reported study found that, whilst small corpora may not be large enough to train IE systems in their own right, augmenting such corpora with training instances derived from other corpora can help to improve the performance of the trained system. This provides convincing evidence that combining smaller, richly annotated corpora, such as our own, with larger corpora which are slightly poorer in information content, could provide a future direction of research for training more accurate biomedical IE systems. This idea is especially attractive, given that the production of large, richly annotated corpora can be very time consuming.

In the remainder of this paper, we firstly cover the key aspects of our annotation scheme, followed by a description of the recruitment and training of annotators. We follow this by providing detailed statistics, results and evaluation of the GREC, and finally present some conclusions and directions for further research.

## Methods

This section is concerned with the preparatory work required prior to the annotation of the GREC. Beginning with a clarification of our notion of an event, we then provide a description of the key features of our annotation scheme. A brief overview of the annotation software used and of its customisation is followed by details regarding the annotators and their training. As performance during training was measured quantitively through the calculation of inter-annotator agreement (IAA) scores following each cycle of training, we provide details and motivation for our chosen evaluation metric, the *F-measure*. Finally, we provide an analysis of the IAA results attained during training.

### Events in biomedical texts

In this section, we clarify our notion of an event. Firstly, we provide some simple examples of event instances that relate to gene regulation and expression within biomedical texts:

1) *In Escherichia Coli*, *glnAP2 may be*** activated*** by NifA*.

2) *Our results show that glnA ***encodes ***glutamine synthetase*.

For each event, two types of information may be specified in the text, both of which are important to its correct interpretation:

• The participants (or *arguments*) of the event. In sentence 1), there are 3 arguments specified by the verb *activated*, i.e., *In Escherichia Coli*, *glnAP2 *and *NifA*.

• Higher level information (called *modality*) about how the event should be interpreted. For example, the word *may *in sentence 1) indicates that there is some uncertainty about the truth of the event, whilst the phrase *Our results show that *in 2) indicates that there is experimental evidence to back up the event described by *encodes*. Several recent articles (e.g., [47-51]) have reported on attempts to annotate information such as certainty, evidence or negation within biomedical texts.

Our current work concerns the first of these information types. Specifically, the annotation task consists of the following, in sequence:

a) Identifying relevant instances of events that relate to gene regulation and expression.

b) Identifying all arguments of the event that are specified within the same sentence.

c) Finally, assigning semantic roles and biological concepts to these arguments.

Table 1 illustrates the type of information that would be annotated for sentences 1) and 2) above.

**Table 1:** Example annotation output

**Verb**	**AGENT**	**THEME**	**LOCATION**
activated	NifA: *Activator*	glnAP2: *Gene*	In Escherichia Coli: *Wild_Type_Bacteria*

encodes	GlnA: *Gene*	glutamine synthetase: *Enzyme*	

For each verb, its separate arguments within the sentence are indicated, together with the biological concepts assigned to them. Each argument is also categorized according to the semantic roles assigned.

### Annotation scheme

In this section, we outline some key aspects of the annotation scheme. Firstly, we describe the types of events on which the annotation is focussed, i.e., gene regulation and expression. This is followed by a more detailed description of the semantic roles and biological concepts assigned to event arguments. Finally, we provide an account of some of the steps taken to ensure the consistency of annotated text spans.

To accompany the annotation scheme, we have produced a detailed set of annotation guidelines, as these are necessary to aid in the achievement of high quality annotations [52-54]. The structure and content of these guidelines were iteratively refined in discussion with domain experts and with annotators (via group discussion sessions following annotation training phrases and full annotation cycles). The guidelines are available to download from , and are also available in Additional file 1.

### Gene regulation and expression events

The current annotation effort is concerned with events relating only to gene regulation or expression, i.e., events that describe any interaction which leads, either directly or indirectly, to the production of a protein. Annotation is restricted to sentences that contain some mechanical description of transcription, translation or post-transcriptional modifications and/or their controls.

Annotators were helped by (but not restricted to) a list of verbs which we created that potentially denote gene regulation and expression events. These are automatically highlighted by the annotation software, WordFreak (see *Software *section below), in each abstract to be annotated. The basis of this list was a set of 229 hand-picked gene regulation verbs provided by the European Bioinformatics Institute (EMBL-EBI). We augmented this list through the automatic extraction of further verbs from an *E. coli *corpus. This corpus contains approximately 33,000 MEDLINE abstracts and was also provided by EMBL-EBI. The automatic extraction was carried out by identifying those verbs whose syntactic arguments corresponded either to terms identified by the TerMine tool ()or biological named entities identified by the GENIA tagger [55]. The complete list was subsequently reviewed for relevance by a biology expert, resulting in a list of 353 verbs.

### Semantic roles

Starting with the generic semantic roles proposed for VerbNet [29] and PropBank [30], we examined a large number of relevant events within MEDLINE abstracts, in consultation with biologists. We concluded that arguments of gene regulation and expression events may be characterised using a subset of these general language roles, with the addition of the domain-specific CONDITION role. In some cases, we changed the names of the roles used in other resources in an attempt to make them more easily understandable to biologist annotators.

From VerbNet, we have used the roles AGENT, THEME, INSTRUMENT, LOCATION, SOURCE and DESTINATION. Our RATE and TEMPORAL roles are based on the VerbNet EXTENT and TIME roles, respectively. MANNER and PURPOSE come from PropBank's set of general roles that are applicable to any verb.

We also saw a need for a role similar to the VerbNet PREDICATE role, to deal with cases such as:

*The (cAMP)-cAMP receptor protein complex ***functions ***as **an activator***...,

where *an activator *corresponds to the PREDICATE role of *functions*. For our own purposes, we created 2 separate roles, DESCRIPTIVE-AGENT and DESCRIPTIVE-THEME, and extended the characterisation of these roles to apply not only to predicatives, but also to any argument which describes characteristics or behaviour of either the AGENT or the THEME of the event.

The full set of roles is shown in Table 2. In general, definitions of argument types normally specified as adjuncts, such as MANNER, INSTRUMENT, CONDITION and LOCATION, can be problematic to distinguish from each other. However, our use of more biologically-oriented definitions for these cases aims to reduce discrepancies.

**Table 2:** Semantic roles

**ROLE NAME**	**DESCRIPTION**	**EXAMPLE** (**bold** = semantic argument, *italics *= event central verb)
AGENT	Drives/instigates event	**The narL gene product ***activates *the nitrate reductase operon

THEME	a) Affected by/results from event b) Focus of events describing states	**recA protein **was *induced *by UV radiation **The ptsH mutant ***lacks *HPr

MANNER	Method/way in which event is carried out	cpxA gene *increases *the levels of csgA transcription by **dephosphorylation **of CpxR

INSTRUMENT	Used to carry out event	EnvZ *functions *through **OmpR **to control NP porin gene expression in E. coli.

LOCATION	Where *complete *event takes place	Phosphorylation of OmpR *modulates *expression of the ompF and ompC genes in **Escherichia coli**

SOURCE	Start point of event	A transducing lambda phage was *isolated *from **a strain **harboring a glpD"lacZ fusion

DESTINATION	End point of event	Transcription is activated by *binding *of the cyclic AMP (cAMP)-cAMP receptor protein (CRP) complex to **a CRP binding site**

TEMPORAL	Situates event in time/w.r.t another event	The Alp protease activity is *detected *in cells **after introduction **of plasmids

CONDITION	Environmental conditions/changes in conditions	Strains carrying a mutation in the crp structural gene fail to *repress *ODC and ADC activities in response to **increased cAMP**

RATE	Change of level or rate	marR mutations *elevated *inaA expression by **10- to 20-fold **over that of the wild-type.

DESCRIPTIVE-AGENT	Descriptive information about AGENT of event	HyfR *acts *as **a formate-dependent regulator**

DESCRIPTIVE-THEME	Descriptive information about THEME of event	The ptsH mutant *lacks*** HPr**.

PURPOSE	Purpose/reason for the event occurring	The fusion strains were *used*** to study** the regulation of the cysB gene

For each semantic role, a brief description is given, together with an example sentence containing an instance of the role. In the example sentences, the verb on which the event is centred is indicated in italics, whilst the event argument corresponding to the appropriate semantic role is indicated in bold.

### Biological concepts

Our biological concept labels are organised into hierarchies based on the Gene Regulation Ontology (GRO) [43]. This ontology, which integrates and builds on parts of other established bio-ontologies, such as Gene Ontology [56] and Sequence Ontology [57], is also included within the list of ontologies of the OBO Foundry [58]. Our biological concept labels are arranged within 5 different hierarchies, corresponding to the following supercategories: *Nucleic_Acids, Proteins, Living_Systems, Processes *and *Experimental*.

During annotation, biological concepts are identified within each semantic argument. In each case, the most specific concept category possible within the appropriate hierarchy is assigned, based on the context in which the concept occurs. The aim of this is to allow queries over extracted event instances to be performed at different levels of granularity, i.e., users could specify more general or less general concept types according to their requirements.

Consider the following example:

*To map the regulatory domain of Escherichia coli T-protein*...

Here, it is possible to assign the specific concept category *Domain *(within the *Proteins *hierarchy) to the concept *the regulatory domain*, which is a functional part of a protein. However, the following example presents a greater challenge:

*IHF may inhibit ompF transcription by altering how OmpR interacts with the ompF promoter*.

Here, *IHF *is clearly a repressor. However, the specific category of *OmpR *is ambiguous from the context of the sentence between:

• An activator of *the ompF promoter*.

• A repressor of *the ompF promoter*.

Therefore, the more general category of *Regulator *is assigned to *OmpR*.

### Consistent annotation of text spans

The task of annotating consistent text spans is often challenging [52], but is important to ensure a cleanly annotated corpus which is easy to understand, reuse and process. Consider the following sentence:

*The Klebsiella rcsA gene ***encoded ***a polypeptide of 23 kDa*

The AGENT of *encoded *may be viewed as any of the following spans: *Klebsiella rcsA, Klebsiella rcsA gene*, or *The Klebsiella rcsA gene*. Similarly, the THEME could be *polypeptide*, *a polypeptide *or *a polypeptide of 23 kDa*.

In order to promote consistent choice of spans, we have created a number of guidelines which are mostly based on syntactic chunks. Prior to annotation, chunks are automatically identified by the GENIA tagger [55]. The example below illustrates the output of the tagger, in terms of the chunks identified. Note that, according to the output of the GENIA tagger, PP chunks contain only the preposition, and not the following NP.

[NP The Klebsiella rcsA gene] [VP encoded ] [NP a polypeptide ] [PP of ] [NP 23 kDa]

According to our guidelines, annotated text spans should normally consist of (sequences of) complete chunks, thus alleviating many issues relating to the exact words that should be included within an argument text span. This means that, for example, in the above sentence, the AGENT of *encoded *should be chosen as *The Klebsiella rcsA gene*, as this corresponds to a complete NP chunk.

A further guideline stipulates that argument text spans must consist only of base NP chunks, and that additional descriptive information, usually introduced by prepositions, must be *excluded *from argument text spans. In the above sentence, application of this rule means that the THEME of *encoded *is only the chunk *a polypeptide*, whilst *of 23 kDa *is excluded from the argument text span.

### Software

The annotation of the GREC was performed using a Java-based annotation tool called WordFreak ([59]). The tool is designed to support many kinds of annotation of text documents, and can be adapted to new tasks fairly straightforwardly by producing new Java classes that define the task. Much of the work to customise WordFreak for the current task was carried out by ILC-CNR () in Pisa. The customisation helps annotators to conform to the guidelines in a number of ways. For example, occurrences of biologically-relevant verbs are automatically highlighted. In addition, colour-coding is used to distinguish different types of chunks, whilst certain restrictions are imposed in the tool as regards the types of chunks that can constitute different types of semantic arguments, e.g., ADVP chunks can only be labelled with the MANNER role.

### Annotators and training

Due to the requirement for biological knowledge and complete understanding of the abstracts, annotation was undertaken by 6 biology PhD students with native or near-native competency in English. It was also required that annotators had at least some experience in gene regulation. Linguistic expertise would be acquired through the training programme and through study of the annotation guidelines.

As the annotation was carried out as part of the EC BOOTStrep project (), it was subject to strict time constraints, with the amount of time to complete the annotation work being limited to three months. This time constraint firstly meant that we were unable to recruit annotators who all had a similarly high level of knowledge of gene regulation and expression. In addition, due to the envisaged steep learning curve for annotators, it was decided to devote the majority of the time available to annotator training. The employment of 6 annotators, however, allowed a medium-sized final GREC to be annotated in a relatively short space of time.

Initial training sessions introduced the annotation tool and the task, with a particular emphasis being placed on clear positive and negative examples of gene regulation and expression events. This was considered particularly important for those annotators with less experience in gene regulation and expression. The initial training sessions were followed up by 5 fortnightly cycles, during which abstracts were firstly annotated by the annotators and then examined by 2 of the authors (one with biological expertise and the other with linguistic expertise), who produced individual feedback reports for each annotator prior to the start of the next cycle of annotation. Additional regular group sessions allowed problems to be discussed in more detail. The calculation of IAA scores after each training cycle provided a quantitative measure of improvement during the training period. Prior to providing these scores, we first describe our method of calculating agreement.

### Calculating inter-annotator agreement

We have defined a novel evaluation methodology which calculates IAA for a number of separate subtasks of the annotation process. These subtasks are as follows:

• Event identification (how frequently annotators agree on which events to annotate).

• Argument identification (for agreed events, how frequently the same arguments are chosen by each annotator). For this task, we calculate separate agreement rates corresponding to:

*a. Relaxed *span matches, where argument text spans identified by a pair of annotators at least overlap with each other, but do not necessarily match exactly.

*b. Exact *span matches, where argument text spans identified by a pair of annotators match exactly. This statistic helps us to evaluate the effectiveness of our rules for consistent span annotation.

• Semantic role assignment (for agreed arguments, how often the same semantic roles are assigned by each annotator).

• Biological concept identification (within agreed arguments, how often annotators identify the same biological concepts).

• Biological concept category assignment (for agreed biological concepts, how often the assigned categories are agreed upon by each annotator). For this task, we calculate 3 different agreement rates, i.e.,

*a. Exact *category matches, where each annotator has assigned exactly the same concept label.

b. Matches *including parent*, where we also consider as matches those cases where the category assigned by one annotator is the parent concept of the category assigned by the other annotator.

*c. Supercategory *assignment, where we consider only whether each annotator has assigned a concept within the same top level superclass, i.e., *Nucleic_Acids, Proteins, Living_Systems, Processes *and *Experimental*.

Whilst the Kappa statistic [60] has become a standard way of calculating IAA for classification tasks, it is problematic for most of the annotation subtasks outlined above, as it requires classifications to correspond to mutually exclusive and discrete categories. The only subtask which fits neatly into this category is the semantic role assignment subtask. We have thus chosen to follow [61] in choosing the *F-Score *to calculate IAA, as it can be applied straightforwardly to all of the above annotation subtasks.

The *F-Score *is the harmonic mean of *precision *and *recall *scores, which are normally calculated to compare the performance of an information retrieval or extraction system to a gold standard. For the purposes of calculating IAA, precision and recall between two annotators can be calculated by treating one set of annotations as the gold standard. The F-score is the same whichever set of annotations is used as the gold standard [62].

### Agreement during training

Table 3 reports the changes in the IAA rates as the training period progressed. Four of the cycles (C1 - C4) concerned *E. coli *abstracts, whilst the final cycle (C5) switched to annotation of human abstracts. As the final corpus would consist of both *E. coli *and human abstracts, we wanted to verify to what extent annotation quality could be maintained if the species referred to in the abstracts is changed.

**Table 3:** Inter-annotator agreement during training

**Agreement Type**	**C1**	**C2**	**C3**	**C4**	**C5**
Event identification	58.35	56.01	68.26	77.07	71.94

Argument identification (relaxed span match)	80.45	85.05	91.45	89.39	91.09

Argument identification (exact span match)	61.92	63.98	73.96	79.84	79.17

Semantic role assignment	67.27	75.21	93.91	84.89	86.59

Bio-concept identification	71.35	78.65	78.29	88.55	82.36

Bio-concept category assignment (exact category)	72.34	72.05	71.61	68.84	59.76

Bio-concept category assignment (including parent)	77.53	76.74	75.11	71.58	63.65

Bio-concept supercategory assignment	89.21	89.32	93.45	90.57	84.09

Each numbered column (C1 to C5) displays the IAA results calculated after a particular cycle of training, for a number of separate annotation subtasks. Agreement was calculated between each pair of annotators, and the figures shown in the table are averages amongst all pairs of annotators. Training cycles C1 to C4 were concerned with *E. coli *abstracts, whilst cycle C5 concerned human abstracts

The general trend was for the agreement rates to rise gradually between training cycles C1 and C4. In addition, the discrepancy between relaxed and exact span matches narrowed as the training progressed. For most tasks, the agreement rates peaked at the end of cycle C4, with most agreement levels falling in the range 70% - 90%, which we consider to be acceptable [47].

When the species referred to in the abstract was changed (from *E. coli *to human), this resulted in a drop in agreement rates for most tasks, particularly bio-concept assignment, suggesting that a period of adjustment is required when switching to a new species. Two tasks, however, i.e., semantic role assignment and argument identification, seem more domain-independent, in that the agreement rates stayed constant, or even continued to rise slightly, when the species referred to in the text was changed.

The main exception to the general trend for improvement is in the assignment of biological concept categories, for which there was no discernible improvement during the training period. Differing levels of experience in gene regulation may have caused annotators to vary in their ability to accurately assign fine-grained biological concept categories. However, higher levels of agreement are achieved if we take the hierarchical structure of the concept categories into account, and look at cases where the category assigned by one annotator is the parent of the term assigned by the other. If we map all concept categories to their top level supercategories, then agreement rates of up to 90% (after cycle C4) are achieved.

## Results and discussion

Following the training period, the final annotated GREC was produced. In this section, we provide details, statistics and analysis of this corpus. Following some initial general statistics regarding the corpus, we move on to examine the most commonly annotated verbs and nominalised verbs on which events are centred. Subsequently, we examine in more detail the arguments of events, including an analysis of the numbers of arguments that occur in different events, the distribution of different semantic argument types, and the most commonly occurring patterns of arguments. Biological concept assignment is then covered, including the distribution of the assigned concepts amongst the five different supercategories, together with an analysis of the most commonly assigned concepts. Finally, we consider quality control of the GREC, including both IAA scores and annotator discrepancies that were found through manual examination of the corpus.

### Corpus characteristics and statistics

Candidate abstracts for annotation for the final GREC were selected from species-specific corpora of MEDLINE abstracts collected by EMBL-EBI, who chose abstracts relevant to the *E. coli *and human species using their own rule-based species-filtering methods. The candidate abstracts were further screened for relevance to gene regulation by one of the authors with biological expertise. General statistics regarding the GREC are shown in Table 4. The effort expended by the 6 annotators amounted to a total of 876 person hours (equivalent to 6.4 person months).

**Table 4:** General corpus statistics

	**Complete Corpus**	***E. coli *abstracts**	**Human abstracts**
No of abstracts	240	167	73

No of events	3067	2394	673

Average Events per abstract	12.78	14.34	9.22

Distinct nom. verbs annotated	91	81	36

Events centred on nominalised verbs	1274 (42%)	1066 (45%)	208 (31%)

Distinct verbs annotated	184	152	107

Events centred on verbs	1793 (58%)	1328 (55%)	465 (69%)

Separate figures are shown for the complete corpus, the *E. coli *part of the corpus and the human part of the corpus.

The statistics in Table 4 reinforce the importance of considering events that are described by nominalised verbs as well as those that are described by verbs. In the *E. coli *corpus, events that are centred on nominalised verbs are almost as common as those centred on verbs, although the range of different words that are used to describe events is much greater for verbs than for nominalised verbs.

### Verbs and nominalised verbs expressing events

Table 5 shows the top 10 most common words (verbs and nominalised verbs) which express events, both in the corpus as a whole, and separately for the *E. coli *and human parts of the corpus. In each case, events centred on these 10 words constitute 45 - 50% of the total events annotated, suggesting that the majority of relevant events are centred on a relatively small set of words. Indeed, in the corpus as a whole, only 55 words (either verbs or nominalised verbs) have been used to annotate 10 or more events.

**Table 5:** Most common words describing events

**Combined**		***E. coli***		**Human**	
		
**Event word**	**Count** **(%)**	**Event word**	**Count** **(%)**	**Event word**	**Count** **(%)**
		
Expression N	362 (11.83)	Expression N	309 (12.91)	Expression N	53 (7.88)
		
Encode V	175 (5.71)	Transcription N	139 (5.81)	Encode V	50 (7.43)
		
Transcription N	171 (5.58)	Encode V	125 (5.22)	Express V	36 (5.35)
		
Bind V	143 (4.66)	Bind V	110 (4.59)	Bind V	33 (4.90)
		
Regulation N	119 (3.88)	Regulation N	102 (4.26)	Transcription N	32 (4.75)
		
Activate V	106 (3.46)	Regulate V	87 (3.63)	Activate V	29 (4.31)
		
Regulate V	106 (3.46)	Activate V	77 (3.22)	Interact V	21 (3.12)
		
Repress V	82 (2.67)	Repress V	72 (3.01)	Regulate V	19 (2.82)
		
Require V	73 (2.38)	Binding N	61 (2.55)	Require V	19 (2.82)
		
Activation N	67 (2.18)	Repression N	60 (2.51)	Involve V	18 (2.67)

Separate lists are shown for the corpus as a whole (*combined*), and for the separate *E. coli *and human parts of the corpus. For each word, its type is given (either (V) erb or (N)ominalised verb) together with an indication of the total number annotated events centred on the word and the percentage of all events in the corpus (or corpus part) that this figure represents.

Most of the words in Table 5 correspond to important biological processes. For some of these processes, occurrences of both the verbal and nominalised forms are quite common, e.g., *regulate/regulation, bind/binding, repress/repression, activate/activation*. In other cases, there appears to be a stronger preference for either the verb or the nominalised verb. In the *E. coli *portion of the corpus, for example, twice as many events are centred on the nominalised verb *expression *than any other word. *Transcription *is also rarely used in its verbal form, i.e. *transcribe *(16 times in the complete corpus), whilst *encode *is only ever used in its verbal form.

### Event arguments

**Figure 1:**
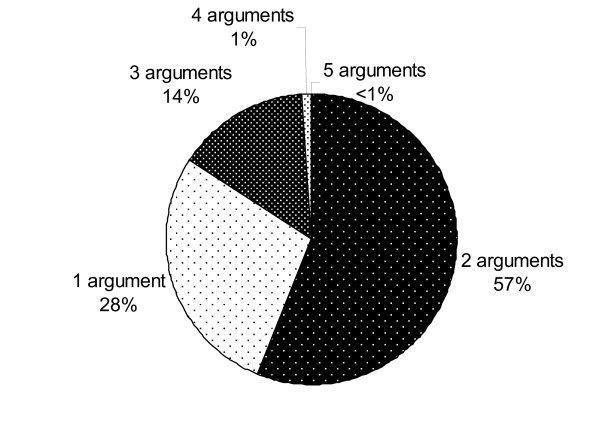
**Distribution of event argument counts**. Each section of the chart shows the percentage of events in the GREC that have been annotated with the indicated number of arguments.

In this section, we provide some statistics regarding annotated event arguments. Firstly, Figure 1 provides an analysis of the numbers of arguments that were identified for different events.

Whilst it is most common for 1 or 2 arguments to be specified, 15% of events specify 3 or more arguments. However, as Figure 1 shows, it is extremely rare in our corpus for 4 or more arguments to be specified. Table 6 provides statistics regarding the semantic roles that were assigned to arguments.

**Table 6:** Semantic role occurrences

**Role**	**Count**	**% events where present**
THEME	2593	84.55

AGENT	1648	53.73

MANNER	416	13.56

LOCATION	300	9.78

DESTINATION	193	6.29

CONDITION	152	4.96

DESCRIPTIVE-THEME	137	4.47

SOURCE	83	2.71

DESCRIPTIVE-AGENT	68	2.22

PURPOSE	65	2.12

TEMPORAL	53	1.73

RATE	50	1.63

INSTRUMENT	32	1.04

For each role, the total number of event arguments to which the role has been assigned in the corpus is indicated, together with the percentage of events in which the role has been assigned to an argument.

In addition to the 13 roles already introduced, there is a further role named *Underspecified*, which was to be assigned by annotators to arguments that could not be characterised by one of the 13 defined roles. However, the fact that the *Underspecified *role was only assigned 11 times in the whole corpus suggests that our originally-defined role set is sufficient to characterise the vast majority of semantic arguments.

The AGENT and THEME roles, which provide the most fundamental information about events, are by far the most commonly assigned. Whilst it may seem surprising that only about half of the events specify an AGENT, this can partly be explained by the relatively high occurrence of events that are centred on nominalised verbs (42% of all events) and passive constructions (14% of events). According to our corpus, only around 20% of events centred on nominalised verbs and 50% of events using passive verb constructions specify an AGENT. Several other roles feature fairly prominently in the events, particularly MANNER, LOCATION, DESTINATION and CONDITION, which is in line with observations made by [20].

In Table 7, the most common patterns semantic roles assigned to event arguments are shown. The most common pattern is for only an AGENT and a THEME to be a specified, constituting almost a third of all events. When events do specify a third argument, it is most common for the AGENT and THEME, plus one additional type of argument, to be present.

**Table 7:** Most common semantic role patterns

**AGENT**	**THEME**	**Other**	**Count (%)**
AGENT	THEME		947 (30.88)

	THEME		693 (22.60)

	THEME	DESCRIPTIVE-THEME	119 (3.88)

	THEME	LOCATION	117 (3.81)

AGENT	THEME	MANNER	113 (3.68)

AGENT		DESTINATION	113 (3.68)

	THEME	MANNER	112 (3.65)

AGENT	THEME	LOCATION	64 (2.09)

AGENT			59 (1.92)

AGENT		DESCRIPTIVE-AGENT	51 (1.66)

	THEME	CONDITION	47 (1.53)

		MANNER	42 (1.37)

AGENT	THEME	CONDITION	38 (1.24)

		SOURCE	36 (1.17)

	THEME	PURPOSE	31 (1.01)

The patterns shown are independent of their ordering in the text. If the roles of AGENT and/or THEME are present in the pattern, this is indicated in the 1^st ^and 2^nd ^columns, respectively. The 3^rd ^column shows any other role present in the pattern (the most common patterns all have a maximum of one role which is not AGENT and/or THEME). The final column shows the total number of events that have been annotated with each pattern in the corpus, together with the percentage of all events in the corpus that this figure represents.

### Biological concepts

**Figure 2:**
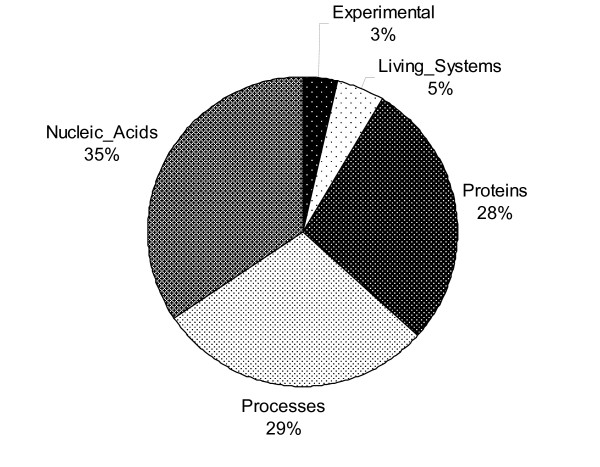
**Distribution of biological concept supercategories**. Each section of the chart shows the percentage of annotated biological concepts in the GREC that have been assigned a concept class belonging to the indicated supercategory.

In the corpus as a whole, 5026 biological concepts were identified. The distribution of the categories assigned to these concepts amongst the five supercategories is shown in Figure 2.

The supercategories *Nucleic_Acids *and *Proteins *are so dominant because most gene regulation and expression events describe some kind of relationship between entities of these two types. The *Processes *supercategory is also very common, as concepts assigned to this correspond to "embedded" events that describe a mechanistic link between *Nucleic_Acid *and *Proteins*, e.g.:

**Expression ***of the ompF and ompC genes is ***affected ***in a reciprocal manner by the osmolarity of the growth medium*.

Annotators were instructed to assign the most specific concept possible in the hierarchy; the results show that 66.91% of assignments indeed constitute the most specific concepts. Table 8 compares the most commonly assigned concepts in the *E. coli *and human parts of the corpus.

**Table 8:** Comparison of concept assignments in *E. coli *and human abstracts

***E. coli***			**Human**		
	
**Category**	**Count** **(%)**	**Type**	**Category**	**Count** **(%)**	**Type**
	
Gene	645 (16.41)	G	Gene	129 (11.77)	G
	
Gene_Expression	350 (8.91)	G	Protein	112 (10.22)	S
	
Regulator	287 (7.30)	S	Transcription_Factor	107 (9.76)	G
	
Promoter	255 (6.49)	S	Gene_Expression	83 (7.57)	G
	
Transcription	200 (5.09)	S	Cells	61 (5.57)	S
	
Regulation	199 (5.06)	S	Transcription	60 (5.47)	S
	
Gene_Activation	189 (4.81)	S	Gene_Activation	60 (5.47)	S
	
Protein	170 (4.33)	S	Activator	47 (4.29)	S
	
Repressor	158 (4.02)	S	Regulation	43 (3.92)	S
	
Activator	150 (3.81)	S	DNA	33 (3.01)	S
	
Operon	148 (3.77)	S	Promoter	31 (2.83)	S
	
Gene_Repression	136 (3.46)	S	Transcription_Binding_Site	31 (2.83)	G
	
Locus	99 (2.52)	S	Protein_Complex	26 (2.37)	S
	
Enzyme	82 (2.09)	G	Sub_Unit	23 (2.10)	S
	
DNA	79 (2.01)	S	mRNA	22 (2.01)	S

Separate lists are shown for *E. coli *abstracts and human abstracts. For each category, the total number of identified concepts assigned to the category is indicated, together with the percentage of all events in the corpus section that this figure represents. The *Type *column indicates whether each category is a *(G)eneral *category within its hierarchy (meaning that it has its own child concepts) or a *(S)pecific *category, indicating that it is a bottom-level category with no child concepts within its hierarchy.

*Gene *constitutes the most commonly assigned concept in both parts of the corpus. It is a general, rather than a specific concept in the *Nucleic_Acids *hierarchy. However, the frequency of assignments of its specific subtypes, i.e., *Mutant_Gene, ORF *and *Allele *is very low, with 19, 10 and 1 assignment, respectively. This suggests that more specific concept type assignment for genes can be problematic.

The category *Transcription_Factor *also has far more assignments than its sub-categories, *Repressor *and *Activator *in the human part of the corpus. However, *Transcription_Factor *is not nearly as frequent in *E. coli *abstracts as in human corpus (see Table 7). These differences represent important biological information: due to the relative complexity of eukaryotic systems, transcription factors play a very important role in gene regulation compared to prokaryotes, like *E. coli*.

### Quality control

Previously, we showed that good rates of agreement were achieved by the end of the training period. To ensure annotation quality was maintained in the final GREC, approximately one quarter of the abstracts was annotated by all annotators. In this section, we firstly present some general agreement statistics relating to the whole corpus, followed by more detailed statistics regarding semantic role and biological concept assignment. Finally, we examine some types of annotator discrepancies that were found through manual examination of the corpus.

### General agreement statistics

Average agreement rates for the final corpus were calculated in the same way and for the same annotation subtasks as during the training period. These are reported in Table 9, categorised according to abstract subject. In most cases, agreement rates are maintain the same level, or in some cases exceed those attained by the end of the training period.

**Table 9:** General agreement statistics in the GREC

**Agreement Type**	**F-Score**	
	***E. coli***	**Human**
	
Event identification	72.27%	76.37%
Argument identification(relaxed span match)	90.23%	91.27%
Argument identification (exact span match)	75.10%	77.48%
Semantic role assignment	88.96%	88.30%
Biological concept identification	82.55%	82.03%
Bio-concept category assignment(exact)	71.02%	66.03%
Bio-concept assignment(including parent)	75.38%	68.97%
Bio-concept supercategory assignment	95.52%	94.75%

Average F-Score agreement figures are shown for several annotation substasks, with separate figures being shown for the *E. coli *and human parts of the corpus.

Particularly high levels of agreement (88% or above) are achieved for both the identification of semantic arguments and the assignment of semantic roles to these arguments. As these are the subtasks that we originally identified as being more linguistically-oriented than others, our results suggest that a detailed set of guidelines, together with an intensive training programme, allow these tasks to be carried out by biologists to a high degree of accuracy.

### Semantic role assignment

Table 10 provides more detailed agreement rates for semantic role assignment. High levels of agreement (over 84%) are achieved amongst many of the most commonly occurring roles, including AGENT, THEME, MANNER, LOCATION, DESTINATION and SOURCE. However, CONDITION and DESCRIPTIVE-THEME are also fairly common, but have lower rates of agreement. Discrepancies have been examined and are further discussed in the *Annotator Discrepancies *section below. Most of the other role types occur much less frequently in the corpus (varying between 1-5% of events), meaning that the agreement rates shown may be less reliable.

**Table 10:** Individual role agreement statistics

***E. coli***			**Human**		
	
**Role**	**N**	**F-score**	**Role**	**N**	**F-score**
	
THEME	5560	92.41%	SOURCE	10	100%
	
AGENT	3702	92.31%	LOCATION	302	96.36%
	
MANNER	697	86.68%	AGENT	2009	92.95%
	
DESTINATION	486	85.42%	DESTINATION	403	92.12%
	
SOURCE	250	84.71%	MANNER	344	90.84%
	
LOCATION	425	84.25%	THEME	2485	89.67%
	
RATE	176	76.44%	PURPOSE	53	89.47%
	
CONDITION	227	67.26%	TEMPORAL	41	72.00%
	
PURPOSE	85	41.95%	CONDITION	21	58.82%
	
DESCRIPTIVE-THEME	259	39.72%	DESCRIPTIVE-THEME	234	57.46%
	
DESCRIPTIVE-AGENT	100	34.32%	DESCRIPTIVE-AGENT	90	36.36%
	
TEMPORAL	33	25.00%	INSTRUMENT	9	0.00%
	
INSTRUMENT	9	16.5%	RATE	5	0.00%

Separate statistics are shown for the *E. coli *and human parts of the corpus. Within each part, semantic roles are ordered according to their agreement rates. The columns headed *N *show the total number of assignments for each role. Assignments by each pair of annotators are counted separately and added to the total.

### Biological concept assignment

Whilst Table 9 showed that coarse-grained biological category assignment achieved around 95% agreement, the assignment of finer-grained categories achieved the lowest agreement rates amongst all annotation subtasks. Table 11 shows the most commonly assigned categories in each portion of the corpus, together with their agreement rates.

**Table 11:** Individual biological concept category agreement statistics

***E. coli***			**Human**		
	
**Category**	**N**	**F-score**	**Category**	**N**	**F-score**
	
Gene	2010	90.55%	Gene	432	89.35%
	
Protein	771	51.88%	Protein	419	61.58%
	
Promoter	644	95.34%	Transcription_Factor	301	51.83%
	
Repressor	436	68.35%	DNA	298	63.08%
	
Operon	434	85.25%	Promoter	154	92.21%
	
Gene_Expression	407	78.62%	Transcription_Binding_Site	140	50.00%
	
Regulator	349	25.21%	Transcription	118	100.00%
	
Activator	345	42.32%	Cells	111	95.49%
	
Locus	192	72.91%	Regulation	66	96.97%
	
Enzyme	176	89.77%	Activator	65	9.23%

Separate statistics are shown for the *E. coli *and human parts of the corpus. Within each part, categories are ordered according to their total number of assignments, as shown in the columns headed with *N*. Assignments by each pair of annotators are counted separately and added to the total.

Table 11 illustrates that there are several differences in the most commonly assigned concepts according to the species referred to in the abstract (i.e., *E. coli *or human). There are also large differences in the rates of agreement for different categories, which are not correlated with their frequency of occurrence. High levels of agreement (over 75%) are achieved for a number of these categories, most notably *Transcription, Cells, Regulation, Promoter, Gene *and *Enzyme*. In general, the classes with the highest agreement seem to be those that do not have very specific interpretations, i.e., those concepts with broader interpretations which are understandable by biologists with different backgrounds. This means that the highest levels of agreement have been reached when the context dictates that a very specific concept cannot be assigned. Less agreement is achieved for categories that are more specific to the context of gene regulation and expression, such as *Activator*, *Repressor*, *Transcription_Factor*, etc.

### Annotator discrepancies

Certain discrepancies between annotators exist in the final corpus, of which a number are highlighted in this section. Whilst the identification of these discrepancies will help to refine the guidelines for future phases of annotation, it was also found that certain errors were being made which were already covered in the guidelines. Thus, there may be a need to more carefully balance conciseness with comprehensiveness in the guidelines.

### Event identification

The majority of discrepancies in event identification concern nominalised verbs. A particular example is the word *mutation*, which can be used either as a nominalised verb (i.e., the action of mutating), or as an entity (e.g., a mutated gene). However, the distinction can sometimes be problematic. Consider the following examples:

1) *In addition, the pleiotropic phenotypes conferred by **a particular envZ mutation **(envZ473) required the presence of functional OmpR protein*.

2) *Therefore, OmpF reduction resulted in **a mutation **in the marA region*.

In sentence 1), *a particular envZ mutation *seems to describe a mutated entity rather than the action of mutation. In contrast, the *mutation *in sentence 2) describes the action of *marA *being mutated due to reduction of *OmpF*.

### Argument identification

Argument identification discrepancies often occurred in more complex sentences, in which a "double layer" of annotation was sometimes required. In the following sentence, *Alpha interferon *should be seen as the AGENT of *converting *as well as the AGENT of *stimulates*:

***Alpha interferon ****stimulates transcription by ***converting ***the positive transcriptional regulator ISGF3 from a latent to an active form*.

LOCATION arguments can also be problematic in sentences containing multiple events. In the following sentence, for example, different annotators associated the LOCATION *in Escherichia coli K-12 *with either the event described by the verb *control *or the nominalised verb *expression*.

*EnvZ functions through OmpR to ***control ***porin gene ***expression *in Escherichia coli K-12***.

### Semantic role assignment

Amongst the most important semantic roles, both in terms of frequency of occurrence and according to [20], CONDITION is the one with the lowest rates of agreement. We thus examined more closely the types of disagreements that occur. According to our study, the most common confusions are with the MANNER and TEMPORAL roles. Typical examples include the following:

1) *In contrast, the ****anaerobic *repression ***of ethanol dehydrogenase by nitrate does not require the narL product*.

2) *Nitrate repression, however*, **was significantly enhanced ***(sevenfold) when **the cells were cultured in minimal medium***.

For the *repression *event in sentence 1), *anaerobic *was confused between MANNER and CONDITION. The confusion may occur because *anaerobic *can be used in the description of environmental conditions in a phrase such as *under anaerobic conditions*. Here, however, it is being used to describe the *method *of repression, and hence the MANNER role is most appropriate. In sentence 2), the phrase *the cells were cultured in minimal medium *was annotated either as a CONDITION or as a TEMPORAL argument of the *enhanced *event. Whilst this would normally be interpreted as a CONDITION, the confusion may have arisen due to the use of *when *at the beginning of the phrase.

Regarding the DESCRIPTIVE-THEME role, the most common type of confusion is with THEME. According to the guidelines, one of the situations in which DESCRIPTIVE-THEME should be assigned is to objects of verbs that describe states rather than actions e.g.,

*The fru operon ***contains ***the genes for IIFru*.

Here, there is no action and hence no AGENT. Thus, *the fru operon *is the THEME and *the genes for IIFru *is the DESCRIPTIVE-THEME.

However, problems sometimes arose for certain verbs such as *exhibit*, where there may be some confusion as to whether a "state" or "active" interpretation should be taken, e.g.,

*The wild-type and mutant ompR genes ***exhibit ***different phenotypes of osmoregulation*...

The interpretation taken by the annotator determines whether *different phenotypes *is assigned the role THEME or DESCRIPTIVE-THEME (and also whether *The wild-type and mutant ompR genes *is assigned AGENT or THEME).

In general, DESCRIPTIVE-THEME and DESCRIPTIVE-AGENT have less strict definitions than other roles, in that the only restriction imposed is that they should be assigned to arguments that describe characteristics or behaviour of the AGENT or THEME. This, together with the fact that they are not particularly commonly occurring, could have made them more difficult to assign accurately. As future work, we will consider tightening the definitions and possibly splitting them into different roles. Although it is desirable to keep the set of roles used as small and as general as possible in order to ease the burden on the annotator, a slightly larger range of more tightly-defined roles may help to improve agreement rates.

### Biological concept assignment

As observed in Table 11, there is much more discrepancy between certain biological concept categories than others, especially those that constitute context-specific concepts. An exception to this is *Protein*, which is a more general concept category within the *Proteins *supercategory. We therefore examined the most common concept categories with which *Protein *was confused. These are shown in Table 12.

**Table 12:** Most common concept categories confused with *Protein*

***E. coli***		**Human**	
	
**Category**	**N**	**Category**	**N**
	
Regulator	108	Transcription_Factor	74
	
Activator	87	Activator	27
	
Repressor	59	Regulator	16
	
Transcription_Factor	29	Gene	9
	
Gene	27	Sub_Unit	8

Separate statistics are shown for the *E. coli *and human parts of the corpus. Within each part, the categories are ordered according to the number of times that the confusion occurred, as indicated in the columns headed *N*.

With the exception of *Gene *(which belongs to the *Nucleic_Acids *superclass), all other categories confused with *Protein *are also categories within the *Proteins *supercategory. This suggests that some annotators were using the *Protein *category to encompass all things related to proteins, rather than assigning more specific category labels. This may be related to their differing levels of knowledge regarding gene regulation and expression.

## Conclusion

We have designed an event annotation scheme for biomedical texts and produced an associated corpus, the GREC, consisting of 240 MEDLINE abstracts annotated with 3067 gene regulation event instances. The corpus is unique within the biomedical field in that it combines both linguistically-oriented features (i.e., event-independent semantic roles tuned to the domain) and biologically-oriented features (i.e., biological concepts linked to the Gene Regulation Ontology [43]).

The corpus can act as a basis for creating domain-specific semantic frame resources, and has already been used in the production of semantic frames for inclusion within the BioLexicon [44], in which the semantic frames are linked with syntactic information. It is also hoped that the corpus will boost research into other areas of bio-IE, such as the production of domain-specific SRLs, which have previously suffered due to the lack of a suitably annotated corpus. Initial experiments have demonstrated the feasibility of training an SRL using the corpus and as such, we hope to exploit the corpus in future shared tasks with such an aim There is also evidence to suggest that combining the GREC with other larger biomedical corpora may help to train more accurate IE systems.

Evaluation of the corpus quality was carried out using a newly-devised methodology, taking into account multiple aspects of the annotation task. Average agreement rates for the various tasks fell within the range of 66% - 90% F-score. Through error analysis of the corpus, we identified the most problematic issues, which included difficulties in assigning particular semantic roles, particularly CONDITION and DESCRIPTIVE-THEME. A full examination of the problematic cases will allow us to further improve the guidelines and possibly impose further restrictions in the annotation software, to prevent common types of errors being made.

As regards biological concepts, our results show that, although high levels of agreement can be achieved when considering a coarse-grained set of categories, the use of a fine-grained classification caused some difficulties. This is possibly due to the differing levels of expertise of annotators within the gene regulation and expression domain, which may have resulted in varying levels of confidence in assigning more specific concepts. A solution for further phases of annotation would be to analyze the domain knowledge of annotators in greater detail and, where appropriate, provide extra training in the assignment of more specific categories. This may be combined with a re-evaluation and possible simplification of the concept hierarchies.

A further major direction of future work will be to apply our scheme to a greater range of biomedical texts that describe a wider range of event types. Whilst other event types may require the use of alternative biological concepts or ontologies, we would like to verify that our set of semantic roles is applicable to events in other areas of biomedicine. The texts we will consider will also include full texts, in which events may be expressed in different ways from abstracts, and may involve different (higher) numbers of arguments.

Finally, we wish to ensure that others can use and evaluate the GREC as simply as possible. Our future plan includes facilitating this in two different ways:

Firstly, in response to the current diversity of corpus annotation formats and the problems this causes in their comparative evaluation [63], a shared format has been created for resources for biomedical relation extraction [15], together with a standard for the evaluation of relation extraction methods using this data [64]. We plan to convert our own corpus to this format, which has already been carried out for several biomedical corpora, e.g., [13,16,21,23].

Secondly, we plan to develop a corpus reader which will allow the GREC to be made available within the U-Compare system [65](). This is an integrated text mining/natural language processing system based on the UIMA Framework [66], which provides access to a large collection of ready-to-use interoperable natural language processing components.

## Authors' contributions

All authors contributed to the production of the manuscript. SA supervised all steps of the work. PT and JM designed the linguistic aspects of the annotation scheme, whilst SAI was responsible for the biological aspects of the scheme. JM coordinated the recruitment of the annotators. PT and SAI produced the annotation guidelines and supervised the annotation process, including the training of the annotators and the production of feedback reports. PT designed the evaluation scheme. All authors read and approved the final manuscript.

## Supplementary Material

Additional file 1 **GREC mini-website**. This website provides brief details of the GREC and descriptions of the available corpus formats. It also provides links to download both the corpus and the annotation guidelines. Click here for file
