# Supplementary material for: Construction of an annotated corpus to support biomedical information extraction
Source: BMC Bioinformatics. 2009 Oct 23;10:349. doi: 10.1186/1471-2105-10-349 (PMC2774701; doi:10.1186/1471-2105-10-349)
Supplement: Additional file 1 — GREC mini-website. This website provides brief details of the GREC and descriptions of the available corpus formats. It also provides links to download both the corpus and the annotation guidelines. [file 1471-2105-10-349-S1.ZIP › Event_annotation_guidelines.pdf]

Event Annotation Guidelines

Contributers:

Paul Thompson, S. Amir Iqbal, John McNaught,
Yutaka Sasaki, Sophia Ananiadou

School of Computer Science, University of Manchester, UK

{paul.thompson, john.mcnaught, yutaka.sasaki, sophia.ananiaou}@manchester.ac.uk
S.Iqbal-2@postgrad.manchester.ac.uk

Simonetta Montemagni, Giulia Venturi

ILC-CNR, Pisa, Italy

{simonetta.montemagni, giulia.venturi}@manchester.ac.uk

© University of Manchester and ILC-CNR

1 INTRODUCTION **3**

2 EVENTS AND VARIABLES **3**

3 NOMINALISED VERBS **4**

4 CONCEPTS **5**

5 THE TASK **18**

6 DETERMINING APPROPRIATE EVENTS TO ANNOTATE **20**

7 IDENTIFYING VARIABLES **21**

7.1 DESCRIPTIVE EVENTS 21
7.2 EVENTS SPECIFYING EVIDENCE OR CERTAINTY LEVEL 23
7.3 NEGATIVE EVENTS 24
7.4 EVENTS SPECIFIED USING NOMINALISED VERBS 25

8 MARKING VARIABLE SPANS **26**

8.1 CHUNKS 26
8.2 GENERAL GUIDELINES 27
8.3 TYPE-SPECIFIC GUIDELINES 29
8.3.1 Entity phrases *29*
8.3.2 Event phrases *32*

9 SEMANTIC ROLES **33**

9.1 DESCRIPTION OF SEMANTIC ROLES 33
9.2 AGENT 35
9.3 THEME 38
9.4 MANNER 41
9.5 INSTRUMENT 43
9.6 LOCATION 44
9.7 SOURCE 46
9.8 DESTINATION 47
9.9 TEMPORAL 47
9.10 CONDITION 48
9.11 RATE 50
9.12 DESCRIPTIVE 51
9.13 PURPOSE 53

APPENDIX A: ANNOTATION PROCEDURE **54**

APPENDIX B : QUICK SEMANTIC REFERENCE ROLE GUIDE **57**

© University of Manchester and ILC-CNR

1 Introduction

We are in the process of building a machine-readable dictionary of biological terms and verbs which can help with automatically finding important facts that are contained within biological texts. This document describes a task called *annotation* which will help us in the construction of suitable dictionary entries for verbs. It begins with an explanation of the types of information that we wish to include within these dictionary entries, followed by a description of the task that will be undertaken to collect this information. Finally, a set of guidelines that explain exactly how the task should be carried out are presented.

2 Events and variables

Verbs typically represent different kinds of events. Details of these events, i.e. the variables that are involved in them, are introduced by a set of phrases that accompany the verb in the sentence. The simple sentence shown in (1) helps to illustrate this.

(1) *The narL gene product activates the nitrate reductase operon*

In (1), there is a verb, *activates,* that is surrounded by 2 phrases i.e. *the narL gene product* and *the nitrate reductase operon.* These phrases can be seen to *belong* to the verb, in that they are used to describe the variables involved in the *activation* event. Each phrase represents a different variable that is involved in the event: the phrase *the narL gene product* represents the thing that *causes* or *drives* the event, whilst *the nitrate reductase operon* is the thing *affected by* the event.

In (1), the phrases that denote the variables of the event correspond to the subject and object of the verb, but it is also possible for verbs to have more than 2 variables associated with them, as shown in (2).

(2) *The LysR-type transcriptional regulator CysB controls the repression of hslJ transcription in Escherichia coli*

In (2), the event is represented by the verb *controls.* As with (1), the subject of the verb, i.e. *the LysR-type transcriptional regulator CysB* is what *instigates* that event. Likewise, the object of the verb, i.e. *the repression of hslJ transcription*, is what is *affected by* the event. In (2), however, there is a further phrase associated with this *controls* event, i.e. *in Escherichia coli.* This indicates *where* the described *control* action takes place.

The above examples illustrate that, when considered at a general level, the same types of variables occur with different types of events. In sentences (1) and (2), for example, we have seen that the subjects of both verbs describe what *causes* or *instigates* the event, whilst the objects describe what was *affected by* or *acted upon* during the event. The general type of information that a particular phrase provides about an event is called its *semantic role.*

Each semantic role has a name associated with it. For example, phrases that describe what instigates or drives are assigned the AGENT semantic role, whilst things that are affected by events are assigned the THEME role. AGENT and THEME are considered as *core* roles, in that they provide what is normally the most important information about the event, and at least one of

© University of Manchester and ILC-CNR

them is present in the descriptions of the majority of events. Phrases corresponding to the AGENT and THEME normally occur in closest proximity to the verb that represents the event.

Other phrases can provide a number of other types of information about event, including where the event took place, i.e. the LOCATION role. These phrases normally occur further away from the verb, but are also relevant to the description of the event. Altogether, we have defined 13 semantic roles that seem to characterise the majority of variables involved in biological events. A full list of these roles, together with descriptions and examples, is provided in the section 8 of this document.

Different verbs typically occur with different patterns of variable-denoting phrases. That is to say, the number of phrases that contribute to the description of the event, and the semantic roles that these phrases correspond to, varies from verb to verb. This is because different verbs represent different events, and the description of each event requires a particular set of variables. In order to interpret these events automatically, the dictionary entry for each verb should indicate the patterns of variable-denoting phases that most typically accompany it in biological texts.

3 Nominalised verbs

Whilst events are most typically represented by verbs, it is also possible for them to be represented by nouns. Such nouns are called *nominalised verbs*. They are so called because they convey the same meaning as a related verb, but behave as a noun, in that, for example, they can be preceded by adjectives and/or determiners such as *a* or *the.* Nominalised verbs often have a similar, but different form to their related verbs. **Examples include *transduction* (related verb: *transduce*), *expression* (related verb: *express*), *analysis* (related verb: *analyse*)** Other nominalised verbs have the same form as the verbs from which they are derived, e.g. ***control* and *increase.***

Nominalised verbs are interesting in that, like verbs, they can be accompanied by phrases that correspond to variables involved in the event that they represent. **As nominalised verbs are very common in the biological literature, we want to create similar dictionary entries as for verbs, i.e. to describe the different patterns of variable-denoting phrases that can occur with them.**

In example (2) above, the THEME of the *control* event is actually the description of a further event, i.e. *the repression of hslJ transcription.* The event is represented using a nominalised verb, *repression* (related to the verb *repress*). The THEME of the *repression* event, i.e. what is *being repressed,* follows the preposition *of,* i.e. *hslJ transcription.*

The THEME of the *repression* event, i.e. *hslJ transcription* represents yet another event using the nominalised verb *transcription* (from the verb *transcribe*). In this case, the thing that directly precedes the nominalised verb, i.e. *hslJ,* is a variable in the event. This is the thing that is *undergoing* the transcription, and hence is the THEME of the event.

© University of Manchester and ILC-CNR

4 Concepts

The majority of phrases that denote the variables of events fall into one of two categories:

a) *Things,* such as genes, operons, proteins or species. We refer to these as *entities*
b) *Events,* often expressed using a nominalised verb such as *repression, transcription* or *increase.*

Entities and events can be classified according to the *concept* that they represent, whether this is a gene, species, biological process etc. Part of the annotation task concerns assigning *concept types* to variables, in addition to the more general semantic roles, which were mentioned earlier.

Let us consider again the sentences from section 2.

(1) *The narL gene product activates the nitrate reductase operon*
(2) *The LysR-type transcriptional regulator CysB controls the repression of hslJ transcription in Escherichia coli*

For example, an AGENT was identified for the each of events that were identified in sentences (1) and (2) in section 2 above. However, the *concept type* of the AGENT was different in each case. In the *activates* event of sentence (1), the AGENT was *The narL gene product,* which is a *protein.* On the other hand, the AGENT of the *controls* event in sentence (2), i.e. *The LysR-type transcriptional regulator CysB* represents a different type of concept, i.e. a *regulator.* It may be that in different occurrences of events described by the same verbs, the AGENT can correspond to varying concept types.

In order to assist with the automatic extraction of important facts from biological texts, we would like our dictionary entries to specify the *type(s)* of concepts than can occur as the values of variables. The type(s) specified for each variable will come from a hierarchy of concepts that we have defined for the biological field.

The section headings **PROTEINS**, **NUCLEIC_ACIDS**, **LIVING_SYSTEMS**, **PROCESSES** and **EXPERIMENTAL** are the concept groupings that are used to sub-divide the term-list into a more manageable set of options. Most of these concepts, or classes, are intended to be specific, but unfortunately they are not mutually exclusive. Indeed many can be considered subsets of other classes listed in the term list, such as REGULATOR potentially being a member of PROTEIN_COMPLEX, PROTEIN, BIOLOGICAL_PROCESS.

However, the following general rule applies: **always apply the most specific type from the hierarchy that is applicable to the concept in question.**

In the following section, the hierarchical structure of each group of concepts is shown in the form of a tree, followed by brief definitions of each term. Tree nodes shown in all capitals are for organisational purposes only and do not correspond to assignable concepts.

© University of Manchester and ILC-CNR

PROTEINS

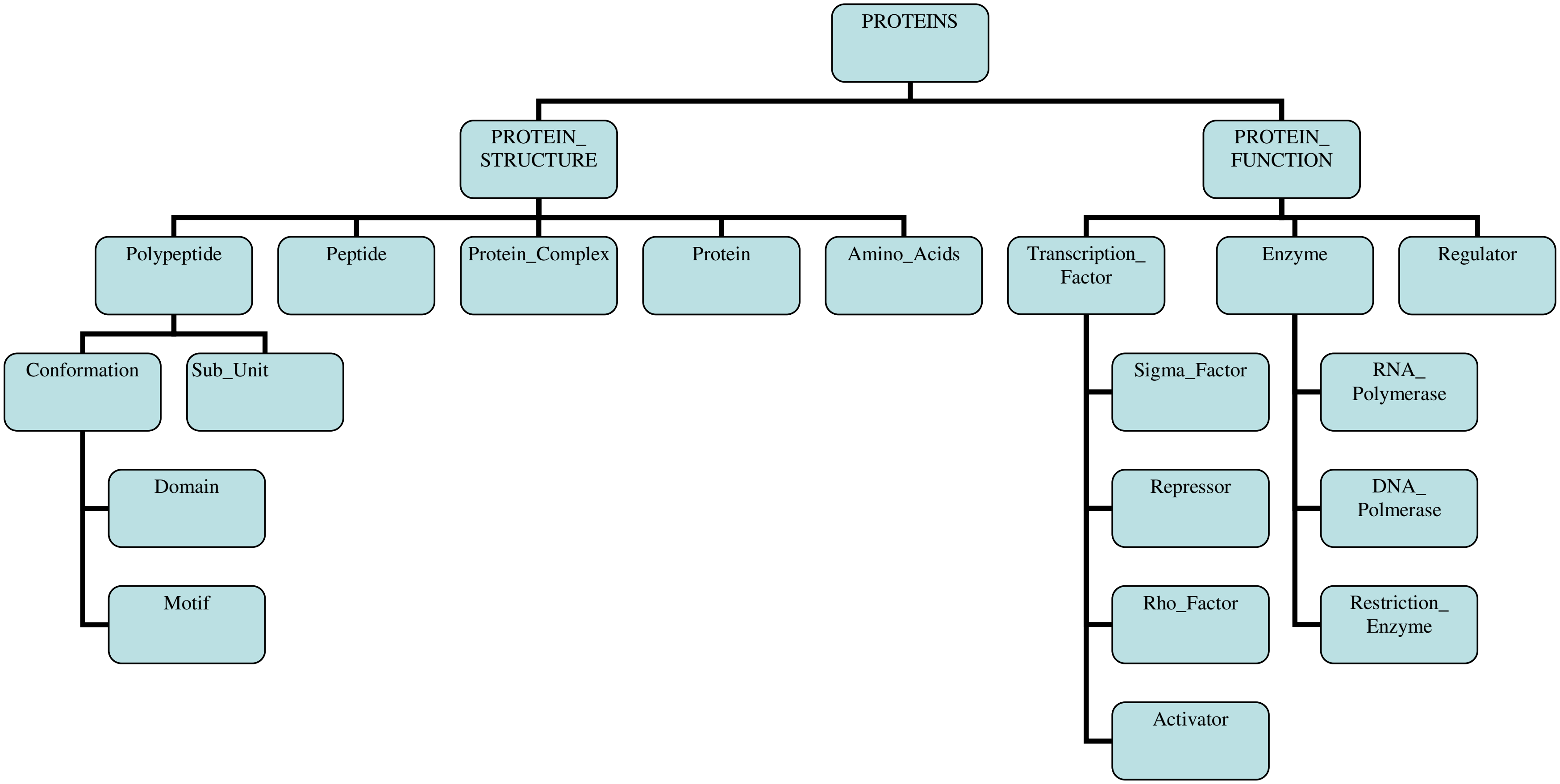

© University of Manchester and ILC-CNR

Proteins

Complex chemical substances chiefly composed of amino acids and their positional references. This includes the physical structure and functional roles associated with each type.

- **Protein_Structure -** specifies the sequence of amino acids that a protein consists of and how these chains of amino acids form a 3-D structure in space. There are 4 levels of protein structure: Primary level is the sequence of amino acids, secondary level is the folding of parts of protein into alpha helix and beta sheets, the tertiary structure signifies the complete conformation of protein in 3-D and quaternary structure is only present when more than one polypeptide chains comprise a protein.

  - **Polypeptide** - A polymer of amino acids usually longer than 50 amino acids. Also identified as protein when it can act alone to perform a biological function.
    - **Conformation -** Refers to the 3-D structure of a polypeptide in space. This is the highest level structure and may also be referred in literature as protein configuration or protein 3-D model.
      - **Domain -** Part of a Protein or Protein_Subunit, usually associated with protein primary structure. e.g. 'superimposable dinucleotide fold domains', 'transmembrane domain II' and assigned a specific function.
      - **Motif -** Motif, or more accurately a structural motif is a local structure in a protein chain which manifests itself as a fold or loop, like 'helix-turn-helix loop'.
    - **Sub-Unit -** A functional part of a Protein which is derived from a process of gene expression independent to the rest of the protein, e.g. 'iron-sulphur protein subunit', 'acyl-carrier subunit'
  - **Peptide** - Short polymer of amino acids containing 2 to 50 amino acids. May not have an associated function or may be a fragment of a protein.
  - **Protein_Complex -** The structure formed by the association of two or more individual polypeptides through non-covalent bonding. A Protein_Complex can perform more than one functions, e.g. include 'The cyclic AMP (cAMP)-cAMP receptor protein complex', 'mutagenic UmuD'C protein complex'.

- **Protein_Function -** This specifies the role of protein in vivo or in vitro. A protein could perform a catalytic role where it is referred to as an enzyme or it may be a part of cell membrane or act as a transport protein. E.g. ATP-ion channel, ATPase dependent pump.

  - **Transcription_Factor -** Any DNA binding protein that binds to a protein binding site on DNA with the aim of regulating gene expression, e.g. 'FabR' or 'RNA polymerase II transcription factor'.
    - **Sigma_Factor** - Any of the reported catalogue of RNA polymerase co-factors, e.g. 'heat shock sigma factor 32', 'eubacterial sigma factor'.
    - **Repressor** - A protein or chemical whose observed effect is to either directly or indirectly <u>lessen or obliterate</u> the rate of gene expression, e.g. 'DNA-binding protein H-NS (represses...)'. Similar verbs would include: 'shrink', 'weaken', 'attenuate', 'ease', 'minimize', etc.
    - **Rho-factor** - A protein found in bacteria which takes part in termination of transcription. It is a part of a much larger termination complex.

© University of Manchester and ILC-CNR

        - **Activator** - A protein or chemical whose observed effect is to either directly or indirectly initiate the process of gene expression, e.g. 'Phosphorylation of OmpF (an activator of...)'. Similar verbs would include: 'start', 'actuate', 'stimulate', 'trigger', 'initiate', 'begin', 'mount', etc.
    - **Enzyme** - All proteins performing catalytic functions are classified as enzymes. The suffix 'ase' is attached at the end of the name of an enzyme to distinguish from other proteins, e.g. 'beta galactosidase' or lactose permease'.
        - **RNA_Polymerase -** An enzyme which transcribes RNA from a DNA template. When the template is RNA, as in some viruses, the enzyme is referred to as reverse transcriptase. All classes of RNA polymerases should be annotated as RNA_Polymerase.
        - **DNA_Polymerase -** An enzyme that is involved in the replication of DNA. Different types of DNA polymerases exist in nature performing polymerization of DNA along with 5' to 3' or 3' to 5' proof reading function. All instances of these enzymes, whether intact enzyme or parts of it should be annotated as DNA_Polymerase. eg. Klenow fragment.
        - **Restriction_Enzyme -** An enzyme that cuts double-stranded DNA at specific sites. The sites are 4 to 8 bp long and are called restriction sites. Examples include 'EcoR1', 'BamH1', 'HindIII' etc.
    - **Regulator** - A protein or chemical whose observed effect is to either directly or indirectly alter the rate of gene expression without a stated bias, e.g. 'FlhD/FlhC (a regulator of...)' where 'a regulator of' is excluded from the span of the term tagged, but used to indicate the type of NER tag used or by the verbs agentive form 'the FlhD/FlhC *regulator'*. Similar verbs would include: 'modulate', 'control', 'govern', 'coordinate', 'guide', etc.

© University of Manchester and ILC-CNR

NUCLEIC_ACIDS

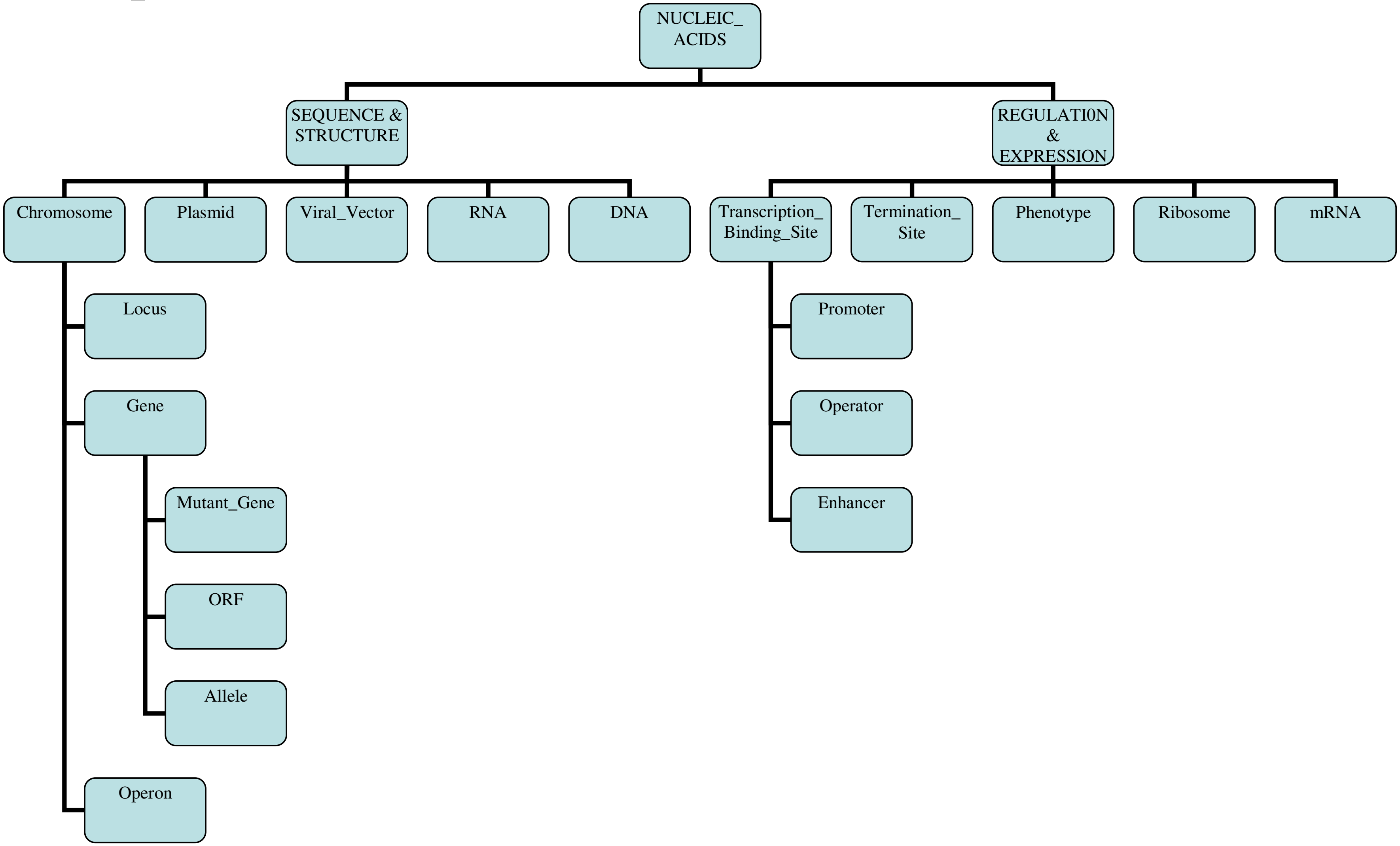

© University of Manchester and ILC-CNR

Nucleic_Acids

- **Sequence & Structure**

  - **Chromosome -** A chromosome is a single long sequence of nucleotides (DNA) which is compacted into a dense structure with the aid of histone proteins. This structure is not visible as such except during mitosis. The two arms of chromosomes are p and q and they should be annotated as Chromosome. In bacteria, the single long chain of genomic DNA is sometime referred to as chromosome and does not contain histone proteins.
    - **Locus**- The reported chromosomal location of a gene, e.g. ‘(at) srl’, ‘(at) recA200’
    - **Gene -** A locatable region of DNA which contains a sequence of bases that encode for the amino acid strings required to build proteins, e.g. ‘lipoprotein gene’, ‘phoE gene’ through the formation of mRNA. Also several genes express rRNA and tRNA.
      - **Mutant_Gene -** Any alteration in the sequence of nucleotides in a gene whether brought on by natural factors or those brought on through the manipulation of the organisms genome, e.g. ‘K-12 lamB mutants’, ‘dnaAts mutants’. The class Mutant_Gene should be applied to all forms of Gene mutation, where the Gene is the term linked to the semantic role.
      - **ORF (Open Reading Frame) -** A region of DNA containing a sequence of bases that could potentially encode a protein, e.g. ‘2754-bp open reading frame’, ‘short-component open reading frames’
      - **Allele-** An allele is an alternative form of a gene (one member of a pair) that is located at a specific position on a specific chromosome. Eg. Blood group A, B and O are allelic forms of a single gene.
    - **Operon -** A functioning unit of DNA composed of an operator, a common promoter, and one or more genes, e.g. ‘lactose operon’.
  - **Plasmid -** A copy of circular DNA found in bacteria and yeast. For example ‘lacZ containing plasmid pBR322’.
  - **Viral_Vector -** A molecule of viral DNA or RNA that is used as a vector for carrying gene segments.
  - **RNA -** Polypeptide of ribonucleotides. For all types of RNA (rRNA, tRNA, siRNA etc.) other than mRNA use the RNA category for annotation.
  - **DNA -** The polypeptide of deoxynucleotides. Any entity comprising of DNA should be assigned to an appropriate category in under the Nucleic_Acids categories. If it cannot be assigned appropriately to any other category, then use DNA category as a last resort. Example ‘the conserved DNA region on chromosome 21’.

- **Regulation & Expression**
  - **Transcription_Binding_Site -** The type associated with a sequence of bases which form the binding sites for proteins involved in the initiation or regulation of gene expression, e.g. ‘TFBS’, ‘TATA binding protein’.
    - **Promoter** -The regulatory region of DNA located upstream of a gene, providing a control point for regulated gene transcription, e.g. ‘promoter of the uxuR’, ‘promoter region’.

© University of Manchester and ILC-CNR

    - **Operator** - A regulatory sequence usually found upstream of an ORF where activator or inhibitor of that gene binds.
    - **Enhancer** - A sequence of DNA found usually upstream of an ORF where an activator protein binds. This results in enhanced expression of the gene.
- **Termination_Site -** A sequence of DNA which identifies the end of a coding sequence.
- **Phenotype -** The observable physical or biochemical characteristics of a living system resulted from the interaction of its genetic makeup and environment.
- **Ribosome -** Any compositional rRNA or subunit structures of a functioning Ribosome.
- **mRNA (messenger RNA) -** The transcribed RNA from an ORF. mRNA in eukaryotes is further processed into introns and exons.

© University of Manchester and ILC-CNR

LIVING_SYSTEMS

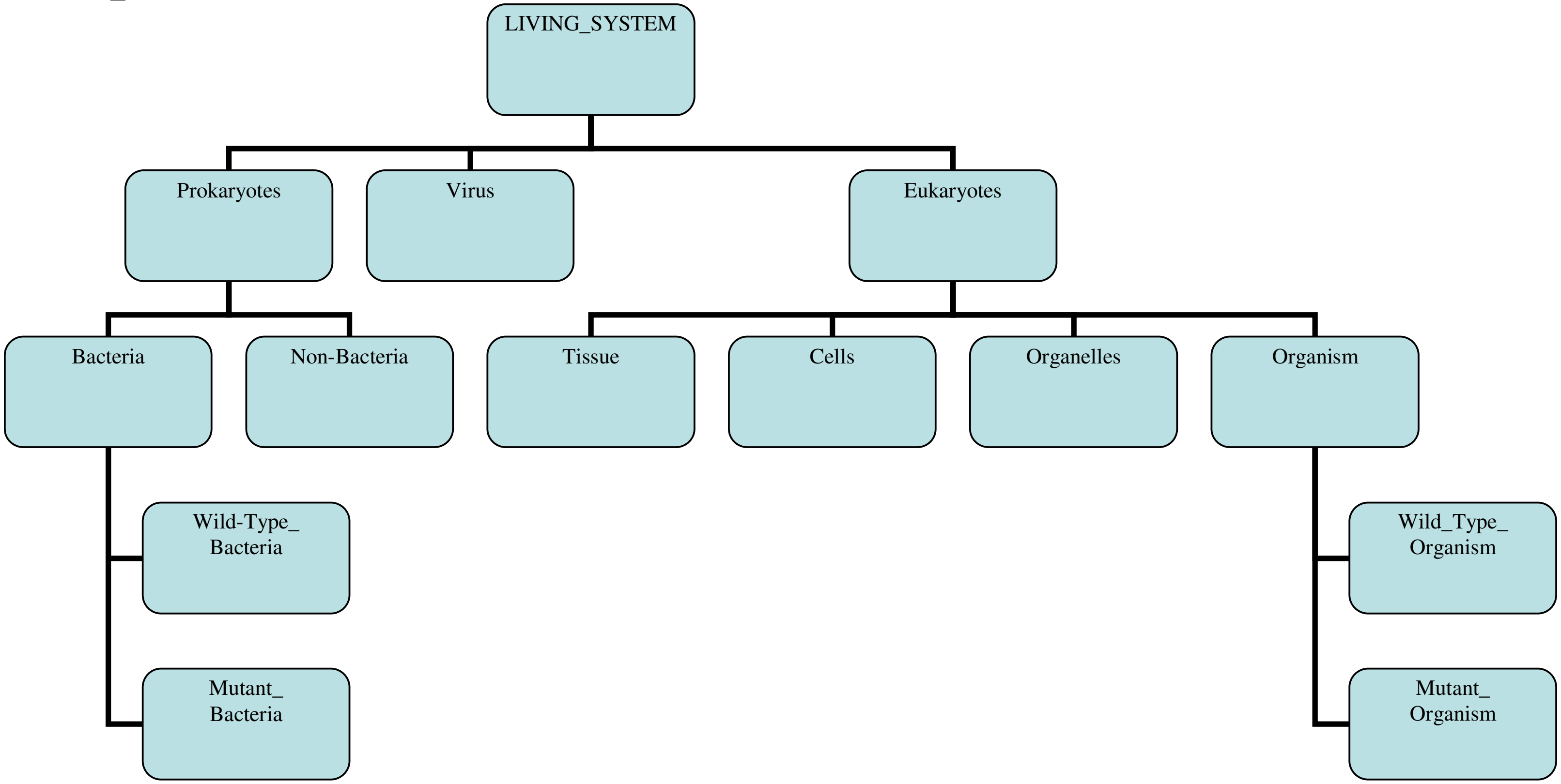

Living_Systems

Here mainly refers to living cells, tissues and organisms.

- **Prokaryotes -** They are a group of organisms, usually single-celled, that lack a nucleus and usually divide through non-sexual binary fission. Examples include bacteria and blue green algae.
  - **Bacteria -** A group of unicellular organisms that are found all over the planet. They are characterized by the absence of nucleus and contain a single long (sometimes circular) DNA molecule. All strains of bacteria should be annotated with category Bacteria.
    - **Wild_Type_Bacteria -** The bacteria found in nature. These are non-modified bacteria found naturally in environment and inside the bodies of living organisms. Only annotate as Wild_Type_Bacteria when the context is clear.
    - **Mutant_Bacteria -** The bacteria whose DNA has been modified structurally by deletion, insertion or point mutation. Only annotate bacteria as mutant when the context specifies it to be so.
  - **Non_Bacteria -** Any single celled prokaryote other than bacteria.
- **Virus -** Virus is an infectious agent that cannot grow outside the body of an organism it infects. Usually referred as virus, eg. Polio virus, ebola virus etc., but could also appear as bacteriophage and as viral vector.
- **Eukaryotes -** These are cells which have distinct nucleus and contain various organelles for specilized functions. All plants, fungi and animals fall into this category.
  - **Tissues -** A complex organization of one or more types of cells. Tissues form the structural basis of organs and systems in complex organisms. Eg. includes bone tissue, muscle tissue etc.
  - **Cells -** The fundamental structural and functional unit of life. Also called the building block of life. Should only be annotated when no choice is found among the other categories.
  - **Organelles -** Subcellular compartmentalized bodies found in eukaryotic cells. Mitochondria and chloroplasts are good examples.
  - **Organism-** It is an individual living system which function independently. The hierarchy of cells and tissues perform specialized functions. All multicellular living systems fall into this category.
    - **Wild_Type_Organism -** An organism that is found in nature. Any organism used in an experiment which is not mutant will go in this category.
    - **Mutant_Organism -** An organism that has been altered genetically to insert or delete a certain function. All transgenic organism like NUDE mice are also included in this category.

© University of Manchester and ILC-CNR

PROCESSES

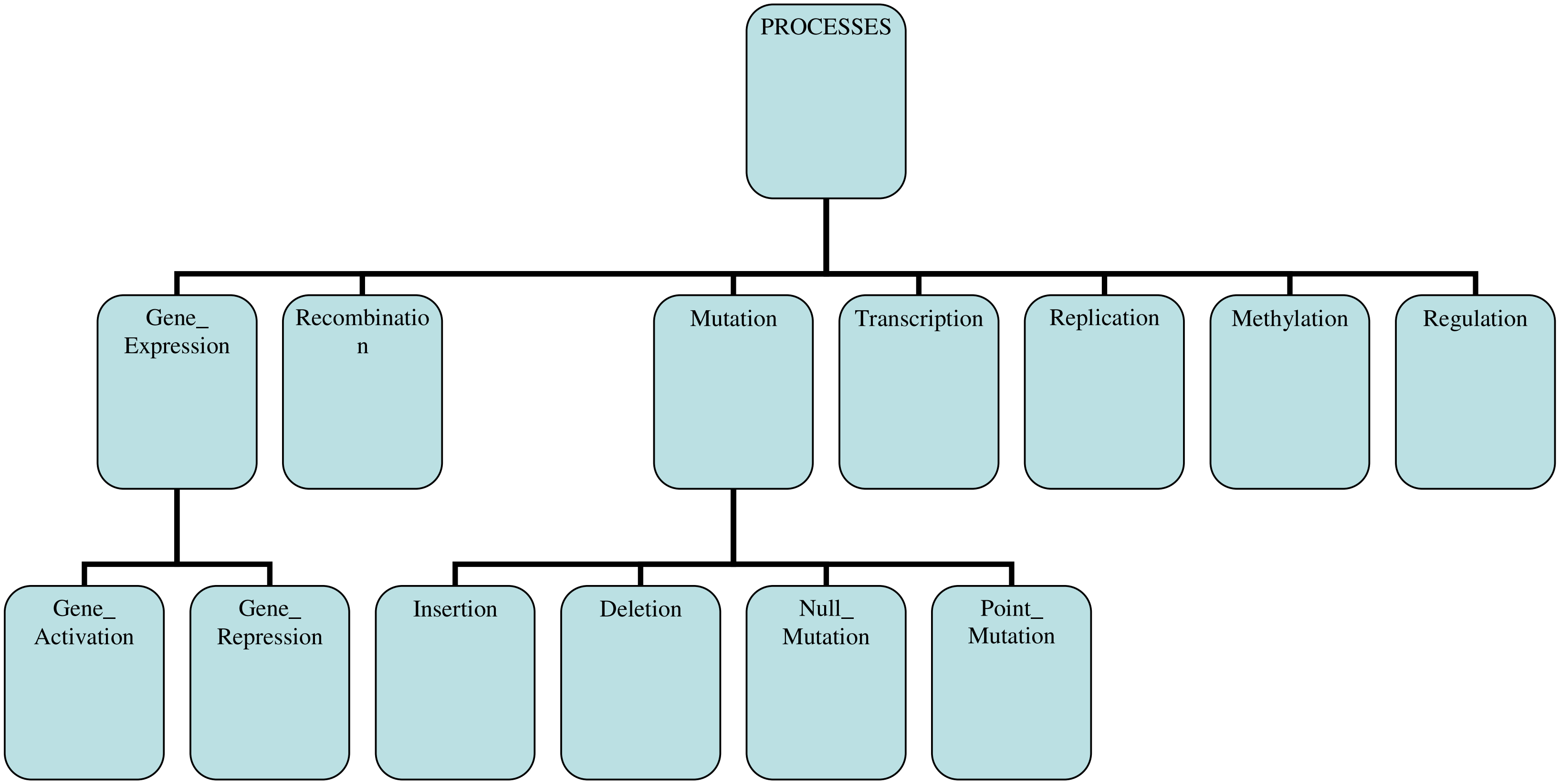

© University of Manchester and ILC-CNR

Processes

A set of concept classes used to label biological processes described in text. The specifics of the interactions described can be inferred from the terms SEMANTIC ROLE TYPES that will be labelled during the document curation process.

- **Gene_Expression -** The process of formation of a protein from a gene.
    - **Gene_Activation -** Implied series of interactions (containing nominalised verbs) ranging from signal transduction leading to the initiation of transcription to the final post-transcriptional modifications of the protein product. In other words, everything to do with the synthesis of a protein, named or otherwise, e.g. 'the gene pathway' or 'kinase activation pathway'
    - **Gene_Repression -** The series of interactions leading to the inhibition of a gene.
- **Recombination -** Process by which genetic material is exchange between the two homologous sister chromatids during synapse formation in prophase stage of mitosis. This term is also used in prokayotes like bacteria when interchange of DNA is taken place.
- **Mutation –** Any alteration in sequence of DNA either by deletion or insertion of nucleotides or through conversion of one nucleotides into other (point mutation). During assigning an event as mutation make it sure the context clarifies the type of mutation. If it is not clear from the context then assign category Mutation.
    - **Insertion -** Any addition of a single or muliple nucleotide base pairs in DNA sequence.
    - **Deletion -** Any deletion of a single or muliple nucleotide base pairs from DNA sequence.
    - **Null_Mutation -** A mutation that does not affect the phenotype of the living system.
    - **Point_Mutation -** A change in single nucleotide in a DNA sequence. For example 'A' replacing 'G'.
- **Transcription -** The process of RNA formation from a DNA template.
- **Replication** - Specifically DNA replication. The process of copying DNA. Here it applies to both choromosome replication in eukaryotes and plasmid replication in bacteria.
- **Methylation -** Specifically in biological systems refers to the addition of methyl group by enzymes to lysine or arginine of histone proteins in the chromosomes. The methylation has wide implications in gene regulation and transcription.
- **Regulation -** More specifically gene regulation is the control of gene expression through a complex myriad of regulatory genes and regulatory proteins.

© University of Manchester and ILC-CNR

EXPERIMENTAL

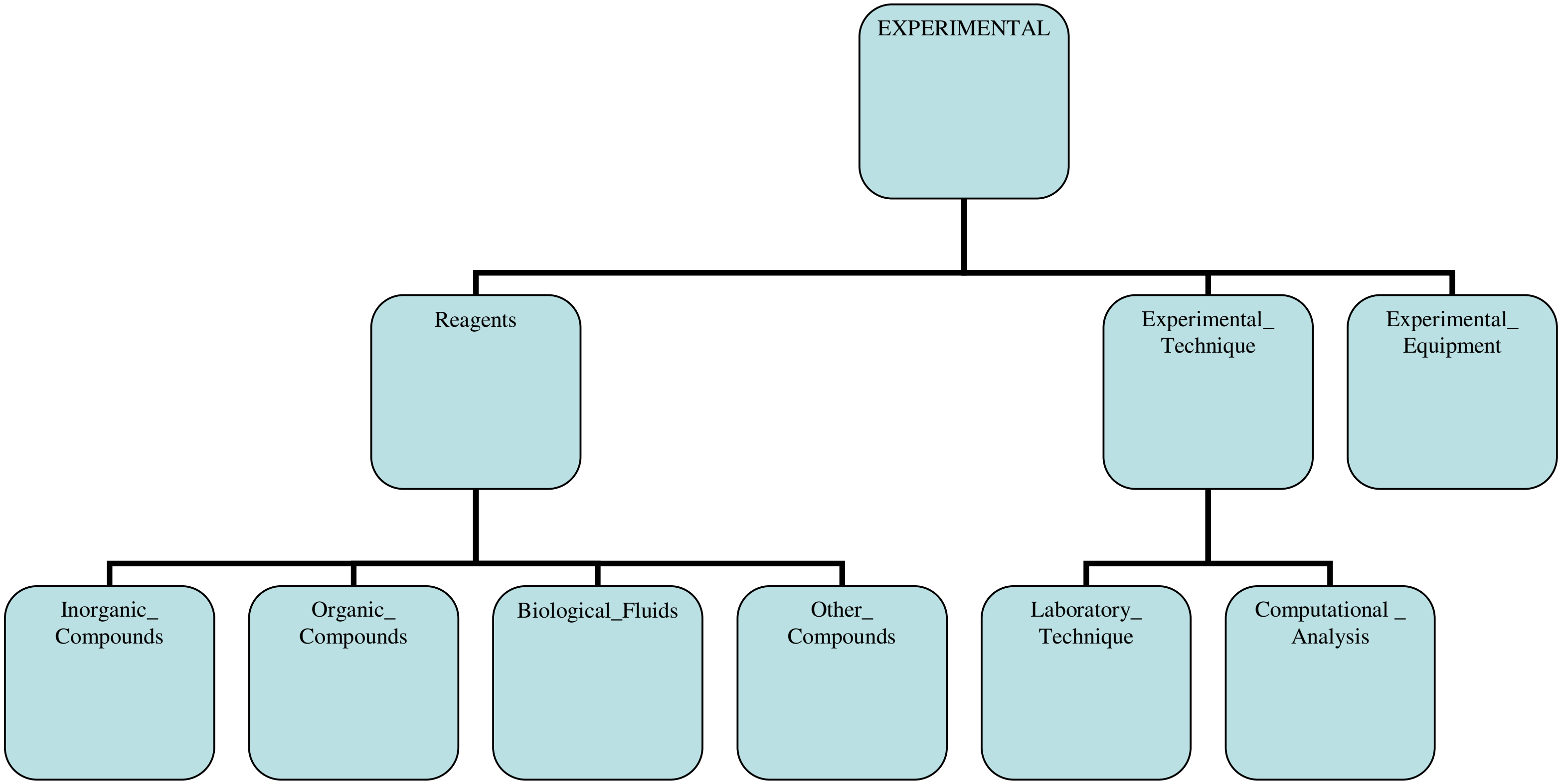

© University of Manchester and ILC-CNR

Experimental

- **Reagents -** Reagent is a general term implied to a chemical substance that is consumed during a chemical reaction.
    - **Inorganic_Compounds -** Non-carbon based compounds such as salts or other minerals.
    - **Organic_Compounds-** For the purposes of this annotation scheme these are the set of carbon based compounds produced through biosynthesis, e.g. lipids, drugs, metabolites.
    - **Biological_Fluids -** Fluids that are present in an organism or in a cell, like blood, lymph and intracellular fluid etc.
    - **Other_Compounds -** Compounds that could not be assigned to any of the above categories. This will be a rare situation as all compounds are organic or in-organic. So use this category when every other option has been exhausted.
- **Experimental_Technique -** The generalised class referring to techniques or SOPs. Most should be covered by either of the two classes 'LABORATORY_TECHNIQUE' or 'COMPUTATIONAL_PROCESS'.
    - **Laboratory_Technique -** The wet-work associated with experimentation. All forms of laboratory-based technique for recording observations, altering conditions or physical forms of the subject under analysis e.g. mass spec, western blot, gene splicing, restriction digests, etc.
    - **Computational_Analysis -** *In-silico* analysis e.g. BLAST homology search, all forms of statistical analysis and any of the dry work associated with experimentation.
- **Experimental_Equipment -** Laboratory equipment used in the execution of experimentation, e.g. laboratory consumables, machines, glassware, etc

© University of Manchester and ILC-CNR

5 The task

We have identified a list biologically-relevant verbs which *potentially* describe gene regulation events. Firstly, we want to discover which of these verbs *actually* represent such events in biomedical abstracts, as well as discovering any further verbs (as well as nominalised verbs), which are not on the list, but which also describe gene regulation events. For each verb that represents a gene regulation event, we would like to construct dictionary entries that characterize their behaviour. In order to do this, we need to discover the following:

a) The *patterns* of variable-denoting phrases that can occur with these verbs.
b) The *semantic roles* of each phrase in the pattern
c) The *type of concept* that best characterizes each phase (if the phrase corresponds to a concept in our hierarchy)

This information will be discovered by examining the usage of these verbs within a large number of biomedical abstracts. For each occurrence in a text of one of the verbs in our list, we wish to carry out a number of steps. This process described is called *annotation*, and will be carried using a program called *WordFreak,* which has been adapted for the task to make it as simple as possible. The tool marks the verbs contained in the biologically relevant list, and allows them to be located automatically. A separate document explains the use of this tool. The steps to be undertaken during annotation are as follows:

1) For each sentence in the abstract, read it carefully and determine which verbs and/or nominalised verbs describe events related to *gene regulation*. The pre-marked verbs are intended to help, but they should only be annotated if they correspond to relevant events. For each relevant verb/nominalised verb identified, the following steps should be undertaken:
2) Identify/locate variables of the event
3) Mark appropriate text spans to represent the event variables.
4) Determine the correct semantic role for each of the variable-denoting phrases identified.

NOTE: All variables that are associated with the event *within the same sentence* should be annotated, regardless of whether one of the existing semantic roles seems appropriate. If none of the semantic roles seems to characterize the variable, then UNDERSPECIFIED role may be applied, together with a comment

5) If the phrase corresponds to a concept in the concept hierarchy (either an entity or a process), assign an appropriate label
6) If a variable-denoting phrase itself a further event, represented using another verb or nominalised verb, then steps 1), 2) and 3) are repeated to identify and characterize the variable-denoting phrases that are used to describe this "embedded" event.

   For example:

   OmpF reduction *required* a mutation in the marA region

   For the verb *required,* the two variables are *OmpF reduction* and *a mutation* both correspond to nominalised verbs. Therefore, they should be annotated as separate events, and their own variables should be identified. *Reduction* has one variable, a THEME, i.e. *OmpF,* whilst *mutation* also has a single variable, a LOCATION, i.e. *in the marA region.*

© University of Manchester and ILC-CNR

NOTES:

a) Nominalised verbs **should NOT as be annotated as separate events** if they specify **no variables of their own**

b) Nominalised verbs **should NOT be confused with other types of nouns that also seem to have variables associated with them**. Nominalised verbs ***only*** correspond to nouns that have the same meanings as verbs (i.e. they represent actions or states). **Some common confusions are as follows**:

   i. "The UV light *inducibility* of the uvrB operon was demonstrated …"
   Here, *inducibility* represents a *property* of the *uvrB operon*, rather than an event, but such properties should ***not*** be marked for their variables. Other examples are *sensitivity* and *absence.*

   ii. "The open reading frame ybbI encodes the *regulator* of expression of the copper-exporting ATPase, CopA."
   Words ending with *–or* or *–er* such as *regulator* correspond to entities perform for a particular action (here an entity that performs *regulation*). Like nominalised verbs, they can have variables associated with them (e.g. what is being regulated). However, such words ***should not*** be considered for annotation.

The basic steps of the annotation task are relatively straightforward. There are, however, a number of challenges to the task, some of which should be made more straightforward by the guidelines that are provided in the sections below. Some of these challenges are as follows:

a) Determining whether each pre-marked verb denotes an event related to *gene regulation.* Annotation should **only** be carried out if this is the case. Other verbs/nominalised verbs should also be annotated, if they describe gene regulation events.

b) Identifying/locating event variables. In many cases, sentences to be annotated can be fairly complex and require careful reading in order to correctly identify the variables.

c) Marking appropriate lengths of variable-denoting phrases. Generally, we want these to be as short and consistent as possible, to aid in the machine-learning of dictionary entries. A set of guidelines provided in the *Marking Spans* section below aims to outline more precisely what should and should not be included within the marked phrases.

d) Assigning appropriate semantic roles to variable denoting phrases. Each semantic role can generally appear in a range of positions or contexts with respect to the verb or nominalised verb that represents the event. The *Semantic Roles* section aims to help with this by providing a clear description of each semantic role, together with examples of different contexts in which variable-denoting phrases corresponding to the role can appear.

e) Determining which verb or nominalised verb a particular variable-denoting phrase belongs to. If there are multiple verbs or nominalised verbs within a sentence, it is important to consider carefully which of these each phrase actually belongs to, i.e. to which of the event descriptions the phase is contributing. Let us reconsider sentence (2) from above:

   The LysR-type transcriptional regulator CysB controls the repression of hslJ transcription in Escherichia coli.

   We determined above that there are 3 events described in this sentence, i.e. *control, repression* and *transcription.* At the end of this sentence is the location *in Escherichia coli*. The challenge is thus the decision of which of the event(s) this location belongs to as

© University of Manchester and ILC-CNR

a variable-denoting phrase. If the location could feasibly apply to more than one event, then it is possible for a variable to be specified as belonging to multiple events.

f) Assigning concept categories to event variable. There are approximately 70 categories, which are organized in a hierarchy. Careful consideration may be required to determine the most appropriate category to assign. It is always the case that the most specific category that can apply to the concept should be assigned. If there is doubt, then a concept further up the hierarchy may be assigned.

6 Determining appropriate events to annotate

As mentioned above, each abstract to be annotated contains a number of pre-marked verbs which have biologically relevant meanings. However, **only those verbs that are relevant to gene regulation should be annotated. In addition, any further verbs or nominalised verbs that are not marked should also be annotated, if they are relevant to gene regulation.**

To put this in clearer terms, the types of events that should be annotated are those that describe any interaction which leads, either directly or indirectly to the production of a protein. This general rule should, however, be restricted to sentences that contain some mechanical description of transcription, translation or post-transcriptional modifications and/or their controls. Some examples include:

- indirect activation of protein production through environmental stimulus
- the finalisation of protein through post-translational modifications including all naturally occuring processes and those manipulated experimentally.
- DNA alterations, mutations, and chimera creation, ***if*** they describe modifications to the process of gene expression or the proteins expressed

Here are some other rules general rules:

- Generally speaking protein-protein interactions are not to be annotated when the result of their interactions does not lead to the expression of a gene.
- Alterations to DNA (structural, compositional), kinetics, that do not lead to gene expression should also **not** be annotated.
- Even if the abstract relies on underlying gene expression, protein finalisation, etc. but does not describe any such reaction in detail, do not annotate. For example, growth of cancerous tissue is obviously the result of aberrant gene expression, but unless the mechanism is described, ignore it.
- Do not annotate events relating to the function of the protein, rather than the processes resulting in the creation of the protein.

For example, in sentence 1 below, both the “binds” and “activates” events should be annotated. However, in sentence 2, the “binds” event should not be annotated, as it is unclear whether or not the interaction leads to an expression event.

1. Protein X binds to Protein Y which activates promoter Z.
2. Gene X expresses Protein Y which binds to the Protein Z.

© University of Manchester and ILC-CNR

However, if Protein Z is described as playing a regulatory role in the same text, then the binding of Protein Y to Protein Z CAN be annotated.

7 Identifying variables

After it has been determined whether a verb relates to a gene regulation event, the next step is to identify the variables involved in the event. **An important point to note here is that variables should be annotated WHETHER or NOT they correspond to biological concepts in the hierarchy. If the variable corresponds to a concept in the hierarchy, then the concept should be assigned. Otherwise, the variable may be assigned only an appropriate semantic role.** For example:

We ***employed*** oligonucleotide-directed site-specific mutagenesis to dissect the promoter region of the gene

For the event denoted by the verb *employed*, the AGENT is *we* i.e. the authors. Although *we* does not correspond to a biologically-relevant entity, it should still be annotated as a variable of the *employed* event and assigned the semantic role of AGENT.

Although identifying variables can often be quite straightforward, the task can be more complex for sentences containing multiple verbs. Normally one of these verbs is referred to as the *main* verb, in that it describes the main or most important event in the sentence, i.e. it characterises what the sentence is about. Other verbs denote secondary events in the sentence.

During the annotation process **it is required that *all* verbs and nominalised verbs that describe relevant gene regulation events (see section 6) are annotated, *regardless of whether these verbs are main verbs or secondary verbs within the sentence*.**

In the following sections, we provide examples and discussion of how variables can be identified in various types of more complex sentences.

7.1 Descriptive events

Sentences sometimes contain descriptive information about an entity or event that is involved in the main event of the sentence. An example is shown in (a).

(a) Expression of the ompF and ompC genes, which *encode* the major outer membrane proteins, OmpF and OmpC, respectively, is *affected* in a reciprocal manner by the osmolarity of the growth medium.

This sentence contains 2 events, namely:
- The main event, denoted by the verb *affected.*
- A secondary event, denoted by the verb, *encode,* which proves descriptive information about some of the entities involved in the main event, i.e. *the ompF and ompC genes.*

© University of Manchester and ILC-CNR

Whenever a sentence contains multiple verbs that are marked for annotation, the ones which relate to gene regulation should all be annotated, regardless of their position in the sentence (i.e. a main or secondary event).

Where there are two or more verbs in a sentence, their variables can be "intertwined" and sometimes well separated from the verb that denotes the event. In (a), for example, the THEME of the *affected* event is the event denoted by the nominalised verb *expression.* This is separated from the verb *affected* by the *encode* event and its variables. It is thus important to think carefully about which parts of the sentence belong to which event. It may be helpful to consider how the different events could be separated out into different sentences, containing the variables associated with a particular event. For (a), this may be done as follows:

i) Expression of the ompF and ompC genes is *affected* in a reciprocal manner by the osmolarity of the growth medium.
ii) The ompF and ompC genes *encode* the major outer membrane proteins, OmpF and OmpC, respectively.

In ii), the word *which* that precedes the verb *encode* in (a) has been replaced by *The ompF and ompC genes*. Verbs preceded by *which* provide a description or extra information about something that has already been mentioned in the sentence; the word *which* is a sort of placeholder for the thing that has previously been mentioned. If the verb following *which* is a verb to be annotated, then it must be determined which other phrase in the sentence the word *which* is referring to: it is this phrase, and not the word *which,* that should be annotated as the variable of the event. In (a), the phrase that *which* is referring to is *The ompF and ompC genes*, an so it is this chunk that should be annotated as the AGENT of the *encode* event.

A final point to note with this example is that the phrase *the ompF and ompC genes* is both the AGENT of the *encodes* event and the THEME of the event denoted by the nominalised verb *expression.* There is no problem with this – the same phrase can be annotated as being a variable of more than one event.

Similar types of constructions can occur with the word *that*, as shown in (b)

(b) Analysis of mutants with deletions that were *derived* from the uxuR::Mud1 insertion strain *confirmed* the counterclockwise transcription direction of the uxuR gene.

In (b), the verb that denotes the main event of the sentence is *confirmed*, whilst a secondary event is denoted by the verb *derived.* This secondary event provides extra information about the mutants. Sentences can be created that contain the variables that are relevant to each event. These are shown in i) and ii).

i) Analysis of mutants with deletions *confirmed* the counterclockwise transcription direction of the uxuR gene.
ii) Mutants with deletions were *derived* from the uxuR::Mud1 insertion strain.

Sometimes, secondary events of the type shown in (b) can be expressed without using *that* but instead using the *–ing* form of the verb. An example is shown in (c).

(c) A mutant strain *displaying* altered regulation of the recA gene was *isolated* as a revertant of a lexA3 recA200 double mutant

© University of Manchester and ILC-CNR

In (c), the variables of both *isolated* (the main verb) and *displaying* should to be annotated. The verb *displaying* is providing a description of the mutant strain; it is a shortened form of *that displays* or *that displayed.* Here, therefore, the phrase *a mutant strain* should be annotated as both the THEME of the *isolated* event and the AGENT of the event denoted by *displaying.*

7.2 Events specifying evidence or certainty level

A certain type of sentence construction is reasonably common when the author wishes to mention explicitly the type of evidence that exists for a mentioned event. An example is shown in (d).

(d) Normal expression of fimA was *shown* to <u>*require*</u> the integration host factor (IHF).

In (d), there are 2 verbs, i.e. *shown* and *require*. Only *require* should be annotated as an event. The main event in this sentence is the one denoted by *require*, whilst the verb *shown* is one of a set of verbs that can be used in this type of sentence construction to indicate the type of evidence for the event. We will refer to these as "evidential" verbs. The verb *shown* indicates that there is strong evidence to back up the specified main event. Replacing *shown* with *believed* would indicate that there is no evidence to back up the *require* event; it would be just a conjecture.

In terms of the structure of sentence (d), the event denoted by *normal expression* belongs to the verb *shown*. However, in terms of meaning, this *expression* event should be marked as a variable of the event denoted by the verb *require,* i.e. the AGENT. This emphasizes the fact that meaning as well as the structure of the sentence should be taken into account during annotation. It is possible to rephrase (d) so that the structure makes it easier to determine that the *expression* event is a variable of the *required* event. This rephrasing is shown in (e).

(e) It was shown that normal expression of fimA <u>*requires*</u> the integration host factor (IHF).

Both the sentence structures shown in (d) and (e) occur in the biological literature. In constructions of the type shown in (d), the subject of the "evidential" verb will almost always be a variable of the verb in the infinitive form (i.e. the one preceded by "to", *require* in (d)). In most cases, it will be the AGENT of the event denoted by the infinitive verb.

A list of "evidential" verbs that can occur in constructions such as (d) and (e) are shown below. This list should be taken as indicative rather than exhaustive.

predict, assume, think, suspect, believe, expect, claim, hypothesise, suggest, claim, indicate, suggest, deduce, argue, infer, show, reveal, demonstrate, confirm, prove, report, find, conclude, observe

NOTE: The majority of the above verbs are purely "evidential" and will not be marked for possible annotation in WordFreak. ***However,*** a small number of the verbs do not have purely evidential uses (i.e. specifying evidence related to another event). The verb *show* is one such example, which can be used to specify an event in its own right. An example is shown below in (f):

© University of Manchester and ILC-CNR

(f) A strain containing a deletion of the sbcB gene <u>*showed*</u> little dRpase activity

Here, *showed* is being used to describe a property of the strain, and hence it should be annotated as an event.

Another construction similar to (d) can occur with verbs such as *seem* and *appear.* These can also be considered as "evidential" verbs, such as the ones in the list above, but occur in slightly different sentence constructions. The construction shown in (e), which is possible for all the verbs shown in the list above, puts the evidential verb in a passive construction, i.e. a form of the verb *to be*, followed by the past tense form of the evidential verb, e.g. *were assumed, was inferred,* etc. However, *seem* and *appear* occur in active constructions. An example is shown in (g).

(g) oxyS RNA *seems to* <u>*modulate*</u> the stability of a region of secondary structure in the ribosome-binding region of the gene's mRNA

Other than the use of the evidential verb in the active form rather than the passive, this sentence behaves in the same way as other ones containing evidential verbs: the subject of the evidential verb is the AGENT of the verb in the infinitive form. Here, therefore, *oxyS RNA* is the AGENT of *modulate.* It can also be rephrased to make the link between *oxyS RNA* and *modulate* clearer, as shown in (h).

(h) It *seems* that oxyS RNA <u>*modulates*</u> the stability of a region of secondary structure in the ribosome-binding region of the gene's mRNA

A final class of verbs that can co-occur with ones that denote events to explicitly indicate the author's level of certainty towards the event are the modal verbs, such as *could, may* or *might.* An example is shown in (i).

(i) Pseudo-HPr activity *could* <u>*replace*</u> HPr in PEP-dependent phosphorylation of PTS carbohydrates.

In (i), Pseudo-HPr activity is structurally the subject of the modal verb *could,* but in terms of meaning, it is also the AGENT of the event denoted by *replace.* Although the sentence expresses uncertainly as to the truth of the event denoted by *replace,* we are not concerned with the truth of the event when performing annotation; we just want to find out the types of variables that can occur with the verb in different contexts.

7.3 Negative events

Following on from what was said in the previous section**, we wish to annotate the variables of events even if the text specifies that the event <u>*did not*</u> happen.** Typically, events are negated using *do* + *not* in active sentences and *be* + *not* in passive sentences. An example is shown in (i).

(j) Several transgenic lines *did* not <u>*express*</u> the lacZ transgene.

© University of Manchester and ILC-CNR

Although (i) conveys the fact that the *express* event did not actually happen, for the purposes of annotation, we consider the event as though it was positive. So, *several transgenic lines* is annotated as the AGENT and *the lacZ transgene* is annotated as the THEME.

Another way in which events can be negated is through the use of the verb *fail,* as shown in (j).

(k) Strains carrying a mutation in the crp structural gene *fail* to <u>repress</u> ODC and ADC activities.

Once again, for the purposes of annotation, the variables of the *repress* event should be labelled with semantic roles as though the event was positive. So, for example, the strains that are the subject of *fail* should be annotated as the AGENT of *repress.*

7.4 Events specified using nominalised verbs

It was described above that variables of events that are denoted by verbs can be further "embedded" events, which are often described using nominalised verbs. In this case, we wish to annotate the phrases that correspond to the variables of the nominalised verb. An example is shown below:

(l) **Phosphorylation** of OmpR by the osmosensor EnvZ <u>modulates</u> **expression** of the ompF and ompC genes in Escherichia coli.

In (k), we initially consider the verb *modulates.* The AGENT and THEME of the event denoted by *modulates* are both "embedded" events that are denoted by nominalised verbs, i.e. the AGENT is the *phosphorylation* event and the THEME is the *expression* event. The verb *modulates* also has a third variable, i.e. the LOCATION of the event, *in Escherichia coli.* Having identified the "embedded" events, we then proceed to identify their own variables. The *phosphorylation* event, for example, specifies a THEME, i.e. *OmpR* and an AGENT, i.e. *the osmosensor EnvZ.* The *expression* event specifies a THEME, i.e. *the ompF and ompC genes.*

It can be noticed that the verb in (k), i.e. *modulates,* acts as a sort of boundary for the variables of the events specified by nominalised verbs. The variables specified of both of these events occur on the same side of the verbs as the event itself. So, both variables of the *phosphorylation* event occur *before* the verb *modulates,* whilst the variables involved in the *expression* event occurs *after* the verb.

When variables involved in events denoted by nominalised verbs are being identified, we impose the restriction that they must *always* occur on the same side of the verb for which the nominalised verb has been identified as a variable. This also applies when phrases on the other side of the verb seem to relate to the nominalised verb, when meaning is considered. An example of this is shown in (l).

(m) **Overproduction** of the exuR repressor also <u>caused</u> **a decrease** of the beta-galactosidase level.

In this example, the variables of the verb *caused* are both events expressed by nominalised verbs. The AGENT is the *overproduction* event and the THEME is the *decrease* event. When

© University of Manchester and ILC-CNR

considering the *decrease* event, the THEME is easily identifiable as *the beta-galactosidase level.* However, if the meaning of the sentence is considered, then the *overproduction* event could be seen as the AGENT of the *decrease* event: the meaning of the sentence is actually that the overproduction of the exuR repressor *decreased* the beta-galactosidase level. However, as the *overproduction* event is separated from the *decrease* event by the verb *caused,* it should *not* be annotated as one of its variables.

8 Marking variable spans

After event variables have been identified, the next step in the annotation process is to mark appropriate text spans to represent each variable. In general, we want these text spans to be as short and consistent as possible. However, determining how much text to annotate can sometimes be a tricky process. In order to help with this, we provide in this section a set of guidelines that aim to help with this consistency by defining the kinds of things that should and should not be included within the marked phrases.

8.1 Chunks

To help generally with consistent marking of phrases, the biological texts are automatically split into *syntactic chunks* before the annotation is begun. Chunks can be considered as the "building blocks" of the sentence, and so it makes sense that these should be the units we consider when determining the variable-denoting phrases to mark. A simple example of a chunked phrase is shown below.

[NP The narL gene product] [VP activates] [NP the nitrate reductase operon] [PP in] [NP Escherichia coli]

In this example, there are 3 types of chunks. NP (noun phrase) chunks contain sequences of nouns, together with any accompanying adjectives and determiners (e.g. *a, the, that* etc). VP (verb phase) chunks contain verbs or groups of verbs that occur together (e.g. *has activated, were activated,* etc.) whilst PP (prepositional phrase) chunks contain prepositions. Other types of chunks that may be identified include ADVP (adverb phrase) which contain adverbs such as *osmotically* or *aerobically.* The three phrases that correspond to the variables involved in the *activation* event are show below:

AGENT: *The narL gene product*
THEME: *the nitrate reductase operon*
LOCATION: *in Escherichia coli*

By comparing these with the chunked text, it can be seen that each variable-denoting phrase is contained within its own chunk. Indeed, it is normally the case that individual chunks, or in some cases sequences of chunks, correspond to event variables. Thus, in order help maintain consistency between different variable-denoting phases, we impose the guideline that variable-denoting phrases should normally consist of whole chunks. This and other guidelines are explained more fully below.

© University of Manchester and ILC-CNR

Instructions of how to correctly select text spans using WordFreak are provided in the WordFreak user manual.

8.2 General guidelines

The guidelines in this section apply to all kinds of event variables.

1) **Normally, phrases that denote event variables should cover complete chunks**.

   Consider the following chunked sentence:

   [NP The Klebsiella rcsA gene] [VP encoded] [NP a polypeptide] [PP of] [NP 23 kDa].

   If we consider what should be the AGENT of the event denoted by the verb *encoded,* there are several stretches of text that could seem appropriate to represent this entity, e.g.

   a) Klebsiella rcsA gene, or just
   b) rcsA

   However, for consistency of annotation, *all* words within the NP chunk should be annotated as the event variable, i.e. *The Klebsiella rcsA gene.* **If possible, the event variable should only span a single chunk.** However, there may be cases where multiple chunks must be spanned in order to fully capture the event variable. Some examples are shown in the more detailed guidelines below.

   In a few special cases, it is permitted for PARTS of chunks to be spanned as event variables. Mainly, this applies to annotating *variables* of *nominalised verbs* which may occur in the same chunk, as detailed below:

   Sometimes, nominalised verbs are directly preceded by an argument, e.g. *hslJ transcription*, where *hslJ* is the thing being transcribed, and hence the THEME of the *transcription* event. In terms of chunking, both the nominalised verb and its THEME occur in the same NP chunk, i.e. *[NP hslJ transcription].* In order to mark *hslJ* as the THEME of the event, it is necessary to use only part of the chunk.

   HOWEVER:

   When a chunk containing a nominalised verb occurs as a variable of another event, then the *whole* of the chunk should be marked as the variable, regardless of whether it contains any variables that belong to the nominalised verb. For example, consider the following sentence:

   [NP marR mutations] ***<u>elevated</u>*** *[NP inaA expression]*

   If we consider the verb *elevated,* then both of its variables are chunks containing nominalised verbs (i.e. *mutations* and *expression*). At this stage of the annotation, the variables should only be considered as “unanalyzed” units. So, the AGENT of *elevated* is

© University of Manchester and ILC-CNR

the chunk *marR mutations*, and the THEME is *inaA expression.* Once the variables of *elevated* have been annotated, their internal structure can be considered, if any of them contain nominalised verbs. In this case, *mutations* has the THEME *marR*, whilst *expression* has the THEME *inaA*.

2) **Annotations may consist of *discontinuous* chunks of text**

It is possible for a single annotation to consist of discontinuous chunks of text, i.e. chunks that are not located next to each other. This may be necessary be comply with some of the more specific guidelines below, where examples are given. Instructions of how to create annotations consisting of discontinuous chunks are provided in the *WordFreak* annotation tool manual.

3) **For most role types, event variables should not begin with prepositions.**

It is often the case that phrases denoting event variables are preceded by prepositions. In most cases, such prepositions should *not* be included within the text span covered by the event variables – although they can be fairly reliable indicators of the semantic role of the phrase, they do not contribute to the meaning of the variable. For example, in passive sentences, AGENTs are preceded by the preposition *by,* as illustrated below.

a) *The polyamine biosynthetic enzymes are negatively controlled [PP by] [NP **cAMP**] in Escherichia coli.*

Here, the event is denoted by the verb *controlled.* In passive sentence such as this, the subject of the verb (in this case *The polyamine biosynthetic enzymes)* is the THEME of the event, whilst the AGENT (*cAMP*) is preceded by the preposition *by.* The fact that *by* precedes *cAMP* is a fairly reliable indicator that it corresponds to the AGENT role. However, *by* does not actually contribute to the *meaning* of the event variable.

Other types of phrases that include prepositions (e.g. *in response to*) may precede arguments playing particular roles, and these should normally also be excluded from the argument text spans. Further details of prepositions and other phrases that typically precede arguments playing semantic roles are found in the descriptions of individual semantic roles in the *Semantic Roles* section below.

4) **Event variables that are assigned the LOCATION and TEMPORAL roles should *always* begin with prepositions, if a preposition is present**

The LOCATION semantic role has previously been briefly mentioned. In contrast to other role types, prepositions that precede LOCATIONs are an integral part of the variable, as they contribute towards its interpretation. Consider the following sentence:

*Dam methylation alters binding of Lrp [PP **at**] [NP **the GATC1130 site**].*

In this example, the preposition *at* does more than just indicating the role played by the phrase that follows. The entity *the GATC1130 site* would be interpreted differently if *at* was replaced by another location-indicating preposition, e.g. *in* or *near.* Thus, for locations, the preposition at the beginning contributes to the meaning of the location, and

© University of Manchester and ILC-CNR

thus should *always* be included within the annotated text span, if present. The same is true for the TEMPORAL role, which is fully section 7.1.8.

8.3 Type-specific guidelines

In the *Concepts* section above, it was described how the majority of phrases that denote variables are either:

a) Entities
b) Events, usually expressed using nominalised verbs, but may also be expressed using another verb

Other categories of phrases, e.g. adverbs, are possible, and are detailed in the descriptions of individual semantic roles in the *Semantic Roles* section where appropriate. The guidelines that follow, however, relate specifically to variable-denoting phrases that correspond to either entities or events.

8.3.1 Entity phrases

Entities can be expressed with various degrees of specificity. Some examples are as follows:

- A general type, e.g. *a positive regulator*
- An name and type, e.g. *the OmpR protein*
- A name only, e.g. *OmpF*

All of these may be marked as event variable phrases in different contexts. However, the general rule that should be followed when marking phrases that correspond to entities is the following:

1) **Only the chunk(s) containing most specific characterization of the entit(ie)s should be marked as the event variable.**

Exactly what constitutes the most specific characterization will vary from sentence to sentence. The most specific characterizations possible are *names* of entities, e.g. *OmpR,* and if chunks containing names are present, then these are the ones that should be annotated. In some cases, entities are referred to only be their names, as in a):

a) *[NP* ***EnvZ****] functions through [NP* ***OmpR****] to control porin gene expression in Escherichia coli K-12.*

In other cases, the entity name is accompanied by its type, but they both occur in the same chunk. In this case, the whole chunk should be marked as the event variable. An example is shown in b).

b) *It was concluded that expression of [NP* ***the uxuR gene****] itself is repressed by its own product.*

© University of Manchester and ILC-CNR

It is often the case that entities represented by names are either preceded or followed by a more general characterization of their type, as shown in c) and d). In such cases, *only* the chunks that contain the name of the entity should be marked as the event variable.

c) *[NP a chromosomal locus], [NP* ***slpA****], ….*
d) *[NP* ***the OmpR protein*** *] , [NP a positive regulator] [PP of] [NP both genes], ….*

If, however, a general characterisation or type of an entity is present *without* an accompanying entity name, then this general characterisation should be marked as the event variable phrase. An example is shown in e).

e) *[NP* ***This operon****] is negatively controlled …*

Sometimes, the name of an entity is accompanied by a shorter name or acronym, often in brackets. In this case, it is the shorter name that should be annotated. Examples are shown in f), g) and h).

f) *[NP the trp promoter] ([NP* ***trpPO*** *]) ….*
g) *[NP the integration host factor] ([NP* ***IHF*** *]) ….*
h) *[NP the fumarate reductase] ([NP* ***frdABCD*** *]) [NP operon].*

It may be the case that the name of an entity spans more than one chunk; in this case, all chunks that contain the name should be spanned, as shown in i) and j).

i) *[NP* ***marA] : : [NP Tn5*** *]*
j) *[NP* ***the uxuR] : : [NP Mud1 insertion strain*** *]*

The next guideline refers to lists of entities:

2) **When list of entities occur, the general rule to follow is that a *single, discontinuous* annotation should be created, consisting *only* of the items in the list, excluding punctuation marks (e.g. commas) and other words such as *and, or* etc.**

An example is shown in k).

k) *A transducing lambda phage carrying [NP* ***glpD''lacZ****], [NP* ***glpR****], and [NP* ***malT****] was isolated from a strain harboring a glpD''lacZ fusion.*

Here, the actual variable annotated consists of the three separate spans *glpD''lacZ*, *glpR* and *malt*, excluding the comma and the word *and.* Instructions of how to create such a discontinuous span can be found in the manual for the *WordFreak* annotation tool. **Concept types should be assigned to each item in the list whether the items represent the same concept or different concepts**. **It is suggested that concept types are assigned to individual entities in the list *prior* to creating the variable annotation.**

As with single entities, lists of entities may be preceded or followed by a general characterization of the entities. The same rule applies about only annotating the most specific characterizations of the entities. In l), m) and n), the general characterizations or long names are followed by shorter entity names, and so, following guidelines 1) above, it is these shorter entity names that should

© University of Manchester and ILC-CNR

be annotated. As with example k), the annotated spans consist of discontinuous chunks, corresponding to the individual items in the lists.

l) *[NP Escherichia coli superoxide dismutase] ([NP **sodA**] and [NP **sodB**]) [NP genes] ....*
m) *[NP the fumarate reductase] ([NP **frdABCD**]) [NP operon] and [NP the aerobic C4-dicarboxylate transporter] ([NP **dctA**]) [NP gene] ….*
n) *The Escherichia coli Ada protein activates sigma(70)-dependent transcription [PP **at**] [NP three different promoters] ([NP **ada**] , [NP **aidB**], and [NP **alkA**]) ….*

In m), the marked entities, i.e. *ada, aidB* and *alkA* specify the LOCATION of the *activates* event. The preposition used to specify the location, i.e. *at,* precedes the more general characterisation of the list of entities, i.e. *three different promoters.* However, according to guideline 4) of the general guidelines for marking entities, prepositions should be included in LOCATION spans if they are present.

Lists of entities that consist only of two items that are conjoined with *and* or *or* many be contained within the same chunk. In this case, a discontinuous span should still be used, by selecting the appropriate parts of the chunk, minus the conjoining word. Examples are shown in o) and p).

o) *[NP the major outer membrane proteins], [NP **OmpF** and **OmpC**]….*
p) *[NP The regulatory proteins **OmpR** and **EnvZ**]….*

In some cases, the full form of lists of items is "reduced", in that a word or phrase at the end of the list applies to all items in the list, for example:

q) the csrB-lacZ expression defects were caused by ***[NP uvrY], [NP csrA], or [NP barA mutations]***

In this case, the list is "shortened", in that the individual items are actually *urvY mutations*, *csrA mutations* and *barA mutations*. In such cases, where the individual items in the list do not have "complete" meanings on their own, the span to be annotated is the ***complete*** span, starting with the earliest item on the list with the incomplete meaning, and ending with the last. In this case, punctuation marks and *and/or* etc, *should* be included within the span annotated.

3) **Negative items in lists should be dealt with in the same way as positive items**

Some lists can include negative as well as positive members, i.e. some members of the list are explicitly marked as not playing the role in the event that the positive members of the list play. Negatively marked items in list are normally preceded *but not*, following the positive items in the list. An example is shown in r).

r) *[NP **Iron**], [NP but not] [NP **manganese**], acted as a corepressor …*

In r), there are 2 items in the list, i.e. *iron* and *manganese*, with *manganese* being negated. In terms of annotation, the list should be treated as though both items are positive, and annotation of the list should proceed according to guideline 2) above. So, the chunks *iron* and *managene* are both annotated as a discontinuous span.

© University of Manchester and ILC-CNR

4) **Only the chunk(s) corresponding to the entity itself, and not any additional information, should be annotated**

Entities are frequently accompanied by extra information or descriptions of some kind, often preceded by a PP chunk. However, only the chunk(s) corresponding to the entity or entities themselves should be marked. The examples s) to u) help to clarify this.

s) *[NP **a transcriptional repressor**] [PP of] [NP Soda]* **…**
t) *[NP **Strains**] [VP carrying] [NP a mutation] [PP in] [NP the crp structural gene]* …
u) *[NP **The uxuA-uxuB operon**] [PP of] [NP the glucuronate pathway]…*

In s), the entity itself is the transcriptional repressor and so this is what should be marked. The chunks following it show what the repressor is acting upon, i.e. *Soda*. This is extra information about the repressor and so should not be marked. In t), the entity itself is just *strains*. The remaining chunks give more specific information about *which* strains are being discussed. In u), the entity itself is *the uxuA-uxuB operon,* whilst the remaining chunks indicate that this operon is part of the glucuronate pathway.

Entities may also be preceded by quantifications (e.g. *some of, many of* etc), as shown in v). These are also considered as extra information and should be excluded from the variable-denoting phrase.

v) *[NP some] [PP of] [NP* ***the novel CsgD-regulated genes***]….

In some cases, when an entity name or general type is not explicitly mentioned, it may be necessary for the variable-denoting phrase to cover several chunks in order to correctly characterise the entity. In w), for example, the THEME of *affects* is not *the arcA modulon* as the THEME of *affects*, but rather *members* of this modulon.

w) *It is possible that Fnr also indirectly [VP affects] [NP some] [PP of] [NP* ***the other members****] [PP* ***of*** *] [NP* ***the arcA modulon*** *].*

8.3.2 Event phrases

A variable involved in an event may correspond to a further event or process. This may be represented using either a verb or nominalised verb, and thus may occur in either an NP or a VP chunk.

1) ***Only*** **the chunk that contains the verb or nominalised verb should be marked as the variable-denoting phrase**.

Chunks that follow the one that contains the verb or nominalised verb may correspond to variables involved in the “embedded” event or process, but these should not be included within the marked variable-denoting phrase. The following examples help to illustrate this.

© University of Manchester and ILC-CNR

a) *[VP **assaying**] the fused lacZ gene product*
b) *[NP **binding**] of the cyclic AMP (cAMP)-cAMP receptor protein (CRP) complex to a CRP binding site*
c) *[NP **The introduction**] of a cysB allele*

9 Semantic roles

Each variable-denoting phrase that contributes to the description of a particular event should be assigned a semantic role. The role labels proposed are general enough to apply to a wide range of variables in different events.

HOWEVER:

In certain cases, it may be that none of the 13 roles defined suitable to characterise a particular event variable. If this is the case, then a 14th role, called UNDERSPECIFIED, may be assigned to an argument. Whenever this role is assigned, it **must** be accompanied by a comment which characterises the role being played by the argument. This will allow us to determine whether further roles must be added to our scheme. *Please also inform us if you encounter such variables.*

9.1 Description of semantic roles

Below are descriptions of our proposed semantic roles. For each role, the following information is provided:

- a general characterisation of the role
- types of arguments that can fill the role (e.g. entities, events, etc.).
- typical clues in the sentence structure or context, e.g. position with respect to the verb or common preceding prepositions. It is important to note that such contexts are only *clues*. Variable-denoting phrases can also occur in other contexts than those detailed; therefore it is always important to consider the general meaning that each variable contributes towards the event, as well as the context, before assigning a role
- a number of illustrative examples. In each example, the verb or nominalised verb of interest is shown in italics, whilst the text span that corresponds to the semantic role being discussed is shown in bold type. The chunking of these text spans is also shown.

Before beginning the description of the semantic roles, there are a number of important points that should be noted.

1) **In certain circumstances, it is possible for a particular semantic role to be assigned to <u>*more than one variable*</u> in an event.**

Consider the following example, where we focus on the nominalised verb *ions:*

*DNase I footprinting assays were used to study the <u>**interactions**</u> of <u>these regulatory proteins</u> with <u>the tsx-p2 promoter region</u>*

There are 2 variables associated with the *interactions* event, i.e. *these regulatory proteins* and *the tsx-p2 promoter region*. **Both of these entities can be seen to be *responsible* for the event occurring, and so it is appropriate that they should both be labelled with the AGENT role.**

© University of Manchester and ILC-CNR

Depending on the meaning of the verb/nominalised verb, it is sometimes possible for 2 *separate* event variables to occur in the form of a *list*. An example is shown below, where we concentrate on the nominalised verb *combinations*:

Complementation was carried out with ***combinations*** *of a host strain and a plasmid.*

The nominalised verb *combinations* is followed by a list of 2 items i.e. *a host strain and a plasmid*. The decision to be made is whether this is a list of items that constitute a *single* event variable, or whether the items in the list represent *separate* event variables.

A simple test to determine this is whether items can be removed without changing the overall meaning of the event (i.e. the event would still make sense). If this is the case, then it is likely that the list corresponds to a single event variable.

A transducing lambda phage ***carrying*** *glpD''lacZ, glpR and malT was isolated …*

In the above example, there are 3 things being carried, i.e. *glpD''lacZ, glpR and malT.* However, if one of more of these items is removed from the list, the event still makes sense, and the overall meaning of the event remains the same (i.e. the lambda phage is carrying *something*). In this case, therefore, the list as a whole corresponds to a *single* event variable.

However, if removing an item from the list changes the meaning of the event, or means that it no longer makes sense, then it is likely that the items in the list correspond to *separate* variables of the event. This is the case for the *combinations* example above. The meaning of this nominalised verb is such that we expect there to be two or more things that are combined together. Hence, if one of the items is removed from the list, then the event no longer makes sense. We can thus conclude that in the above example *a host strain* and *a plasmid* correspond to 2 separate variables of the event. In this case, they are both THEMEs.

2) **The *same* phrase can be annotated as a variable in multiple events.** The following sentence serves to illustrate this:

The LysR-type transcriptional regulator CysB controls the repression of hslJ transcription in Escherichia coli.

In this sentence, it is not easy to determine to which of the three events described (i.e. *control, repression* or *transcription*) the location *in Escherichia coli* applies. Reading surrounding sentences may help to make it clearer which event the location is linked to, but otherwise it is permissible for the phrase to be marked as the LOCATION of more than one event in the sentence.

3) **Care should be taken to determine whether or not a particular phrase constitutes an event variable.**

As a general rule, each annotated variable should contribute a different type of information towards the description of the event. An exception is where 2 or more variables share the same semantic role, as described above. The usual types of information that variables can contribute (i.e. their semantic roles) are described within this section.

© University of Manchester and ILC-CNR

An important distinction to make here is between those phrases that:

a) **actually correspond to event variables**
b) **simply providing extra descriptive information about a variable. In normal circumstances, such descriptive phrases SHOULD NOT be annotated as event variables. An example is shown below:**

Expression of narL *requires* the fnr gene product, a pleiotropic activator.

Here, the event denoted by *requires* has two variables, i.e. an AGENT (the nominalised verb *expression*) and a THEME (*the fnr gene product*). There is additionally additional descriptive information about the THEME, i.e. it is *a pleiotropic activator*. However, descriptive information does not constitute a separate *type* of information relating to the *requires* event. Hence, it is NOT marked as a variable.

An exception to the above rule is when the *event itself* concerns the provision of descriptive information about one of the other event variables. In this case, the variable providing the descriptive information will be assigned either the DESCRIPTIVE-AGENT or DESCRIPTIVE-THEME role (see section 9.1.11). Some examples are shown below:

YjfQ *acts* as a repressor

Here, the purpose of the event is to provide descriptive information about *YjfQ*. Therefore, *a repressor* SHOULD be annotated as a separate variable (in this case, DESCRIPTIVE-AGENT).

This region *contains* a m7G.

Again, the purpose of the *contains* event is to provide descriptive information about *this region.* As such, both the subject and object should be annotated as event variables. In this case, *a m7G* is assigned the DESCRIPTIVE-THEME role.

Where descriptive information SHOULD be annotated as a separate variable, it is normally the case that it is marked with certain prepositions, or it occurs as the object of a verb. More information is provided in section 9.1.11. Other possible cases where descriptive information can be confused with other semantic roles are described in the sections below.

There now follows descriptions of the 12 semantic role types that have been defined for this task.

9.2 AGENT

Below are some general features of variables that correspond to the AGENT semantic role:

- They are ***core* variables**, in that they are very often present, or at least implied, in the description of events.
- They are **responsible for an event occurring**, in that it instigate, drive or triggers the event.
- It follows that **the AGENT role should only be assigned when the event denotes an action of some kind**
- AGENTS are **typically** either **an entity** (see (a)) or **a further event** (see (b))

© University of Manchester and ILC-CNR

- Most **typically**, they occur as **the subject of the verb representing the event** (see (a) and (b))
- In any case, they **normally occur in close proximity to the verb or nominalised verb that represents the event**.

(a) [NP **The narL gene product**] *activates* the nitrate reductase operon
(b) [NP **Phosphorylation**] of OmpR by the osmosensor EnvZ *modulates* expression of the ompF and ompC genes in Escherichia coli

In (b), the marked AGENT phrase occurs further away from the verb of interest, i.e. *modulates*. This is because the intervening phrases correspond to variables involved in the *phosphorylation* event, i.e. the AGENT (*the osmosensor EnvZ*) and the THEME (*OmpR*). Only the chunk containing the word that represents the event, i.e. *phosphorylation,* should be marked as the AGENT of the *modulates* event. The variables involved in the *phosphorylation* event are identified separately.

- **Not all subjects of verbs are AGENTs**. In some cases, events do not have agents at all. This is the case for verbs that describe states rather than actions, where nothing is actually responsible for triggering the event. An example is shown in (c).

  (c) The FNR protein *resembles* CRP (the cyclic-AMP receptor protein)

  The verb *resembles* is not describing an action. Rather, it is used to describe a characteristic of the FNR protein. The protein is not actually *doing* anything as part of this event, and so cannot be responsible for it occurring. Therefore, it should not be classed as an AGENT. In such events, the subject of the verb is normally classed as the THEME; more examples are provided in the next section, where the THEME role is more fully described.

  In passive sentences, it is also the case that the subject will not normally be the AGENT of the event. In a passive sentence, the subject and object are "switched". The verb is in the past tense, and is preceded by a form of the verb *to be,* possibly separated by an adverb, as in (e). An example is shown in (d):

  (d) The transcription of clyA was positively *controlled* by [NP **slyA**]

  In (d), the subject of *controlled* is *The transcription*. However, this is the THEME of the event, as it is what is ***being** controlled* rather than what is ***doing*** the controlling.

- **AGENTs *can* occur in positions other than as the subjects of a verb.** One such case is illustrated in (e).

  (e) The control of uvrB was found to *result* from [NP **direct repression]** by the lexA gene product

  The underlying meaning of this sentence is that the direct repression by the lexA gene product *causes* the control of uvrB. Therefore, it is the repression that is driving the *result* event, and hence *direct repression* should be marked as the AGENT, even though it is the

© University of Manchester and ILC-CNR

object of *results*. This emphasizes the need to carefully consider the meaning of the verb and how the variable-denoting phrases relate to it.

NOTE: Prepositions following a verb can affect its meaning, or at least the interpretation of variables in particular positions. The verb *result* is one such case.

For example:

1) **X results *from* Y**
2) ***X* results *in Y***

In 1), Y is the variable that is responsible for the action, and X is the thing that results. Hence, Y is the AGENT and X is the THEME. However, in 2), the roles are swapped, so that X is the AGENT and Y is the THEME.

- **AGENTs are normally preceded by the preposition *by* in passive sentences**. An example is shown in (f).

  (f) This operon is negatively *controlled* by [NP **the uxuR regulatory gene product**].

  If AGENTs are present in such sentences, they follow the verb, preceded by the preposition *by.*

  IMPORTANT NOTE: The preposition *by* can also precede arguments playing the role of MANNER, which can occur in similar positions with respect to the verb. Care should thus be taken to distinguish between them. Further explanation is provided in the description of the MANNER role (section 9.1.3)

- **AGENTs are often omitted in passive sentences,** i.e. an agent is understood to be causing the action or event, but is not actually specified

  (g) Two types of Escherichia coli were *isolated* and *analyzed* enzymatically.

  In (g), there is no AGENT. The phrase *two types of Escherichia coli* correspond to the THEME of both *isolated* and *analysed.* In both cases, the types of Escherichia coli are the entities affected during the italicised events. Although there is an *implicit* causer of these events (most probably the authors), there is no mention of in this sentence, and hence no AGENT variable is present.

- **Nominalised verbs can also specify AGENTs**. A common way of doing this is shown in (h). The agent follows the nominalised verb, and is preceded by the preposition *by.*

  (h) *Phosphorylation* of OmpR by [NP **the osmosensor EnvZ**] modulates expression of the ompF and ompC genes in Escherichia coli.

- **An event may have more than one AGENT.** This is the case if more than one of the variables in the event can be considered to be responsible for causing the event. An example is shown in (i).

© University of Manchester and ILC-CNR

(i) The results suggest a control circuit whereby [NP **GadW**] *interacts* with [NP **the gadA promoter**].

Here, there are two variables in the *interacts* event, i.e. *GadW* and *the gadA promotor*. When two or more entities interact, they are normally both somehow responsible for the interaction occurring and so in this case, both *GadW* and *gadA* should be assigned the AGENT role.

9.3 THEME

Below are some characteristics of variables that correspond to the THEME semantic role:

- They are ***core*** **variables**, in that they are almost always present
- They are **directly involved in events,** but are **NOT responsible for the events occurring**
- Most THEMES are **entities or further events**
- They normally occur **in close proximity to the verb or nominalised verb that represents the event**

THEMEs can be split into two basic types:

1) In **events describing some sort of action**, denoted by verbs such as *activate, transcribe,* or *induce,* THEMEs correspond to **variables that are acted upon, affected by, or resulting from the event described by the verb or nominalised verb**. In these cases, **the THEME is very often the object of the verb**. Some examples of this type of THEME are shown in (a) and (b). In (a), the THEME is an entity, whilst in (b), it is an embedded event.

(a) The narL gene product *activates* [NP **the nitrate reductase operon**]
(b) Phosphorylation of OmpR by the osmosensor EnvZ *modulates* [NP **expression**] of the ompF and ompC genes in Escherichia coli

2) In **events that describe states**, denoted by verbs such as *occupy, harbour* or *exhibit*, THEMEs correspond to **the "focus" of the event, i.e. the thing whose state is being described**. In such situations, **the THEME is normally the subject of the verb**. Examples of this type of THEME are shown in (c) - (f), where the italicised verbs describe states rather than actions. In these cases, the subjects of the verbs are marked as the THEME, as they cannot be seen to be responsible for the events occurring.

(c) In addition, [NP **the ompR-lacZ fusion**] *exhibits* a dominant OmpR- phenotype.
(d) [NP **The genes**] encoding ribosomal protein S15 (rpsO) and polynucleotide phosphorylase (pnp) *occupy* adjacent positions
(e) [NP **The recA430 protein**] *possesses* ssDNA-dependent rATP activity
(f) [NP **The PhoR1159 protein**] *lacks* the 83 and 158 N-terminal amino acids

© University of Manchester and ILC-CNR

- **In some cases, THEMEs *can* be quite far removed from the verb representing the event.** An example is shown in (g).

(g) [NP **Expression**] of the Escherichia coli torCAD operon, which encodes the trimethylamine N-oxide reductase system, is *regulated* by the presence of trimethylamine N-oxide through the action of the TorR response regulator.

In (g), the THEME of the event denoted by *regulated* is *expression,* although there are a large number of words that separate them. This is because *expression* is followed by a specification of its own theme, i.e. *the Escherichia coli torCAD operon*, after which is a description of this operon, in the clause beginning with *which.* This highlights the importance of reading the *complete* sentence before beginning annotation, in order to gain a full understanding of the event denoted by the verb, and to locate more distant event-denoting phrases.

- **THEMEs can occur in positions other than the object of the verb, even when the verb denotes an action.** An example is shown in (h).

(h) [NP **The control**] of uvrB was found to *result* from direct repression by the lexA gene product

This type of construction was introduced in section 7, where it was stated that the subject of verbs such as *found* will normally be the AGENT of the verb in the infinitive form (in this case *result*). However, the meaning of this infinitive must also be carefully considered in order to correctly assign the roles. In (h), the control of uvrB occurs *in response to* direct repression by the lexA gene product. This means that the *repression* is the AGENT and the *control* is the THEME.

- **In passive sentences, the THEME is normally the subject of the verb**, as illustrated in (i):

(i) [NP **recA protein**] was *induced* by UV radiation

- **In passive sentences, THEMEs should not be confused with AGENTs if the AGENT is omitted.** It is possible for the AGENT of an event to be omitted in passive sentences. If this is the case, care must be taken not to confuse THEMEs with AGENTs. If the verb of interest is in the past tense, and preceded by a form of the verb *to be,* then the subject is normally the THEME rather than the AGENT. An example is shown in (j).

(j) [NP **Two types**] [PP **of**] [NP **Escherichia coli**] were *isolated* and *analyzed* enzymatically.

Here, there are 2 verbs, i.e. *isolated* and *analyzed*, and the THEME of them both is *Two types of Escherichia coli.* The types of Escherichia coli were not *responsible for* the *isolating* and *analysing* events. Rather, they were *affected by* them. The two events were instigated by some unknown agent, presumably human in this case, as experimental methodology is being described.

© University of Manchester and ILC-CNR

- **Be careful of "reduced relative clauses".** In these cases, the verb is in the passive form, but this is not obvious from the surface structure of the sentence. An example is shown in (k).

(k) [NP **The region**] *required* for the activation of putP by CAP was within 234 bp upstream of the translational initiation site

The meaning of the sentence would be more explicit if it began "The region THAT WAS required …". **However, the sentence format shown in (k) requires careful attention to ensure that the correct role of THEME is assigned to *The region.*** By only looking at the structure of the sentence, *The region* looks more like an AGENT.

A further example is shown in (l):

(l) The operator region controls the production of [NP **several proteins**] *involved* in DNA repair, including protein X

The meaning here is that several proteins ARE involved in DNA repair, and hence this NP chunk corresponds to the THEME rather than the AGENT: the proteins are *not* responsible for the involvement.

- **THEMEs are also frequently specified for nominalised verbs**. The most common context in which they occur is after the nominalised verb, preceded by the preposition *of.* In (m), 2 examples of this are shown, with the nominalised verbs *phosphorylation* and *expression.*

(m) *Phosphorylation* of [NP **OmpR**] by the osmosensor EnvZ modulates *expression* of [NP the **ompF** and ompC genes] in Escherichia coli

A further example is shown in (n):

(n) A steep *rise* in the [NP ***synthesis***] of [NP polypeptide] encoded by the model template containing rare codons was demonstrated

In (n), the THEME of *demonstrated* is *A steep rise*. The thing that rose (i.e. the THEME of the *rise* event) was the *synthesis of polypeptide.* As *synthesis* is also a nominalised verb, it is just this NP chunk that gets annotated as the THEME of "rise". A variable of the *synthesis* event is also specified (i.e. *polypeptide).* This is the thing being synthesised, and hence should be annotated as the THEME of *synthesis.*

It is also possible for themes to immediately precede the nominalised verb, within the same chunk. However, as mentioned above, AGENTSs of nominalised verbs may also appear in this position, and so care must be taken that the correct semantic role is assigned. Examples involving THEMEs are shown in (o) and (p).

(o) EnvZ and OmpR act in sequential fashion to activate [NP **porin gene** *expression*].

Here, *porin gene* is the thing that is *being expressed,* i.e. the thing affected by the *expression* event, and hence it should be marked as the THEME.

© University of Manchester and ILC-CNR

(p) The release of 4.5 S RNA from polysomes is affected by antibiotics that inhibit [NP **protein** *synthesis*]

In (p), *protein* is the entity being synthesized (this could be rephrased as *synthesis of protein)* and hence protein is annotated as the THEME.

- **An event may have more than one THEME variable**, as illustrated in (q).

(q) [NP **The coding region**] of the ompF gene was *linked* with the trp promoter ([NP **trpPO**]) preceding ompF.

There are two variables specified for the *linked* event, i.e. *the coding region* and *trpPO*. Note that *trpPO* rather than *the trp promoter* is marked as the second variable because, according to the *Marking Phrases* guidelines, shorter names should be annotated when they are present. The meaning of the event is that the coding region and trpPO were linked together by some unspecified AGENT. They are thus both being affected in some way by the event and so should both be marked with the THEME role.

9.4 MANNER

Variables corresponding to the MANNER semantic role have the following characteristics:

- They describe ***the method or way* in which a particular event is carried out**.
- They are **less central to the basic event description** than THEME or AGENT
- They **Frequently occur further away from the verb or nominalised verb representing the event**
- They should **NOT be confused with the INSTRUMENT semantic role, which corresponds to *entities* used to carry out the event**.

The MANNER role can apply to **a number of different variable-denoting phrases:**

1. **Processes or methods (either biological or experimental) that are employed by the agent to bring about the event**.
   Manners of this sort have the following characteristics:
   - **Normally expressed using verbs or nominalised verbs**, (see (a) –(d)).
   - **Most often preceded by the preposition *by***, but ***via* and *through*** are also possible (see (a) – (c))
   - In some cases, **the verb *using* can also precede MANNER phrases in the same way as prepositions**. (see (d)).
   - **Typically occur *after* the verb representing the event**, as in (a) – (c).
   - **May also precede the verb**, as in (d).

   NOTE: Phrases corresponding to other semantic roles can precede the verb, in the same way as (d).

   (a) cpxA gene *increases* the levels of csgA transcription by [NP **dephosphorylation**] of CpxR

© University of Manchester and ILC-CNR

(b) Transcription of gntT is *activated* by [NP **binding**] of the cyclic AMP (cAMP)-cAMP receptor protein (CRP) complex to a CRP binding site
(c) Structural and functional properties of this regulatory protein were *studied* through [NP **complementation analysis**] of the wild-type and five mutant ompR genes
(d) Using [NP **random Tn10 insertion mutagenesis**], we *isolated* an Escherichia coli mutant strain affected in the regulation of lysU.

Some types of nouns other than nominalised verbs can represent MANNERs, if some kind of action/technique is implied. Examples are shown in (e) – (g)

(e) CsrA *stimulates* UvrY-dependent activation of csrB expression by [NP **BarA-dependent and -independent mechanisms**].
(f) The mechanism underlying feedback inhibition of tufB expression has been *studied* by [NP **gene-dosage experiments**].
(g) Two FadR operator sites of the fadD gene were *identified* at positions -13 to -29 (OD1) and positions -99 to -115 (OD2) by [NP **DNase I footprinting**].

NOTE: An important point to note here is that the same type of phrase can be assigned different semantic roles according to the context and the meaning of the event. For example, *mechanism* is mentioned in both (e) and (f). The context and meaning of the *stimulates* event in (e) means that the phrase is assigned the MANNER role, whilst in (f), *the mechanism* is assigned the THEME role in the context of the *studied* event.

TAKE CARE:

Special care must be taken when assigning a semantic role to phrases preceded by the preposition *by.* This preposition can also be used to indicate AGENTs in passive sentences. The problem arises in passive sentences when the phrase following the preposition *by* refers to some kind of process. This is the case in (b) above. In order to make a decision on the correct role to assign, it must be considered whether a) the *binding* process is what causes the *activation* event to take place, in which case it is the AGENT, or b) the *binding* process refers the way in which the *activation* event is carried out, by some unspecified agent, in which case it is the MANNER.

2. **Adverbs relating to a process that describes how the event is carried out.** Some examples are shown in (h).

   (h) These results suggest that transcription of the fadL gene [VP is **osmotically** *regulated*] by the OmpR-EnvZ two-component system

   Depending on the position of the adverb, it may occur within the VP chunk of the verb that corresponding to the event, or else in a separate adverb chunk (marked ADVP).

NOTE: adverbs should *only* be annotated with the MANNER role if they correspond to a *process* that describes the way in which the event occurs. Adverbs may also correspond to the CONDITION role, as described in section 9.1.9. Other types of adverb, such as

© University of Manchester and ILC-CNR

those that relate to judgements (e.g. *unexpectedly* or *comparatively*) should not be annotated with any type of semantic role.

3. **Certain NP chunks other than those that refer to methods or processes, e.g. NP chunks that end with the word *manner*, or a synonym,** as shown in (i) and (j).

   - These types of manner are **normally preceded by the preposition *in*.**

   (i) Expression of the ompF and ompC genes, which encode the major outer membrane proteins, OmpF and OmpC, respectively, is *affected* in [NP **a reciprocal manner**] by the osmolarity of the growth medium
   (j) These results lead us to conclude that EnvZ and OmpR *act* in [NP **sequential fashion**] to activate porin gene expression; i.e., EnvZ modifies or in some way directs OmpR, which in turn acts at the appropriate porin gene promoter.

4. **Information about the direction of the event**
   - **Expressed either by adverb or NP chunks**, as shown in (k) and (l).

   (k) The gene is *transcribed* [ADVP **counterclockwise**] on the standard Escherichia coli map, as is the uxuAB operon.
   (l) The fhlA gene resides next to the hydB gene at 59 min in the E. coli chromosome, and the two genes are *transcribed* [PP **in**] [NP **opposite directions**].

5. **Fixed set of phrases of latin origin that describe the way in which experiments are carried out.**
   - **These include *in vitro, in vivo, in trans* and *in sys***. Examples are shown in (m) and (n).

   (m) Furthermore, [NP **in vitro** *transcription*] of the fadL gene was strongly repressed by the addition of OmpR and EnvZ proteins.
   (n) *Introduction* [PP **in**] [NP **trans**] of a compatible plasmid carrying a wild-type uxuR gene in the lac fusion plasmid containing strain resulted in a decrease of beta-galactosidase synthesis

9.5 INSTRUMENT

Variables corresponding to the INSTRUMENT semantic role have the following characteristics:

- **They *always* correspond to *entities* that are used by the AGENT in order to carry out the event.**
- **Typically, INSTRUMENTs are preceded by prepositions or other "fixed" phrases.** The most common are ***with, with the aid of, through, using, via*** **or *by.*** Examples are shown in (a) – (c).
- **INSTRUMENTs should NOT be confused with the MANNER semantic role**. Like instruments, MANNERs can be thought of as describing *how* an event is carried out, but MANNERs *never* corresponds to entities.

© University of Manchester and ILC-CNR

(a) We have isolated a strain *carrying* a fusion of the beta-galactosidase structural gene to the promoter of the uxuR regulatory gene with the aid of the Casadaban Mud ([NP **Aprlac**]) phage .
(b) EnvZ VP *functions* through [NP **OmpR**] to control NP porin gene expression PP in NP Escherichia coli K-12 .
(c) Using [NP **MacConkey maltose indicator plates**] we *isolated* an insertion mutation

- **Where the event is denoted using a nominalised verb, it is also possible for the INSTRUMENT to precede the nominalised verb, within the same chunk**. This is the case in (d), where P1 is the entity that is used to carry out the transduction, by some unspecified agent.

(d) [NP **P1** *transduction*] of marA::Tn5 into a Mar mutant partially restored OmpF levels.

9.6 LOCATION

There are **three types of semantic roles that specify information about locations, i.e. LOCATION, SOURCE and DESTINATION**.

- **The LOCATION role is appropriate to assign to phrases that specify where the *whole* event takes place.**
- **They almost always begin with a preposition**
- **In contrast to most other role types, prepositions that occur at the beginning of locations should be included *within* the annotated text span.** This is because prepositions play an important part in the interpretation of locations, as illustrated in the examples that follow.
- **LOCATIONs are normally entities**
- **LOCATIONs can have varying degrees of specificity according to the preposition used.**
- **LOCATIONs should NOT be confused with SOURCE and DESTINATION variables. Such variables can also be considered as locations, but correspond to *start/end* points of events, rather than where the *whole* event takes place.**

<u>**Specific locations**</u>

Locations specified using ***in, on* and *at* are quite specific locations**; they are the actual places in which the event took place. Examples are shown in (a), (b) and (c).

(a) The Escherichia coli Ada protein *activates* sigma(70)-dependent transcription [PP **at**] three different promoters ([NP **ada**], aidB, and alkA) in response to alkylation damage of DNA.
(b) Phosphorylation of OmpR by the osmosensor EnvZ *modulates* expression of the ompF and ompC genes [PP **in**] [NP **Escherichia coli**].
(c) These fusions were *formed* [PP **on**] [NP **plasmid cloning vectors**].

© University of Manchester and ILC-CNR

NOTE: In (a), the preposition *at* does not directly precede the highlighted location. Rather, it precedes *three different promoters.* This is a general characterisation of the location, but a list of more specific entities follows, and we annotate the first of these, according to the *Marking Phrases* guidelines.

Vague locations

The prepositions *near* and *between* specify more vague locations.

In (d), the entity that following the preposition *near* is not the actual location where the event took place. Rather, it is the specification of some entity (in this case rpsL) that is in the *vicinity* of the actual location of the gene. When placed together, the preposition and the entity specify a location, but this is a more vague location than the ones specified in (a), (b) and (c).

(d) The fic gene was *located* [PP **near**] [NP **rpsL**] (formerly strA) on the E. coli K-12 map

In the case of *between,* there are normally two entities that follow. As with *near,* neither of these entities specify the exact location of the mutant. Rather, it is located somewhere in space bounded by these two entities. **The text span covered by locations specified with *between* should cover both entities that specify the bounding points of this location.** An example is shown in (e).

(e) The mutant (alc-24) was *located* [PP **between**] [NP **srl and recA200**] and caused synthesis of high levels of recA protein in both lexA+ and lexA3 strains.

Vague and specific locations

In some cases, 2 locations are specified, i.e. a vague one and a more specific one. **In this case, *both* locations should be annotated as single span assigned the LOCATION semantic role.**

For example, locations on a chromosome may be specified vaguely as being *near* to some other entity, as well as more specifically as the number of minutes on the chromosome. An example is shown in (f).

(f) The gene for ribosomal protein L13, rplM, is *located* [PP **near**] [NP **argR**], [PP **at**] [NP **70 minutes**] on the Escherichia coli chromosomal linkage map.

In this case, *near argR* and *at 70 minutes* should be annotated as a single span, having the LOCATION semantic role.

- **IMPORTANT NOTE: Entities corresponding to locations should normally be assigned a *concept type.*** However, the concept should be assigned ***only* to the entity itself and not to the complete LOCATION span.** For example, the span *in E. Coli* corresponds to the LOCATION variable, but **only the chunk *E. Coli* should be assigned a concept type**

© University of Manchester and ILC-CNR

9.7 SOURCE

Biological events frequently involve a movement or shift from one location to another. The start and/ or end points are locations, but are distinct from the types of location that should be assigned the LOCATION semantic role.

- **The SOURCE role corresponds to phrases that specify where the event *begins.***
- **SOURCE variables normally correspond to entities**
- **They are locations, but should not be confused with the LOCATION role,** which corresponds to where the *whole* event takes place.
- **They are normally preceded by the preposition *from***
- **Unlike LOCATIONs, the preceding preposition SHOULD NOT be included in the annotated span of the variable**

An example of SOURCE role is shown below in (a).

(a) *Transduction* of the marA region from [NP **a Mar strain**], but not a wild-type strain, led to loss of OmpF.

Here, we are focussing on the nominalised verb *transduction.* The THEME of this event, i.e. what is being transduced, is the *marA region*. The marked NP chunk following *from* specifies where the *transduction* event began, i.e. *a Mar strain.* This phrase does not describe where the whole of the *transduction* event took place, and so it is correct to label it as SOURCE rather than LOCATION. A further example is shown in (b), where *a strain* is the start point of the *isolation* event.

(b) To determine the expression of BMI1, a BMI1-LacZ construct was *extracted* from [NP **pBR322 plasmid**] and inserted into E.coli chromosomal DNA.
(c) A transducing lambda phage carrying glpD''lacZ, glpR, and malT was *isolated* from [NP **a strain**] harboring a glpD''lacZ fusion

- **The SOURCE role can also apply to more abstract types of phrases, particularly those with a more "psychological" nature.** An example is shown in (c).

(d) The transcriptional direction of the uxuR gene was *deduced* from [NP **the restriction pattern**] and the phenotypic properties of the new plasmids.

In (c), *the transcriptional direction* is the THEME of the *deduced* event, whilst *the restriction pattern* can be seen as the SOURCE, in an abstract way, as it is a sort of "starting point" of the deduction. Note that *the restriction pattern* is in a list with *the phenotypic properties of the new plasmids* but, according to the *Marking Phrases* guidelines, only the first item in the list is marked as the variable-denoting phrase.

TAKE CARE:

Not every phrase preceded by the preposition *from* constitutes a SOURCE variable.

Consider the example (d)

© University of Manchester and ILC-CNR

(e) That the two divergent transcripts from **the identified promoters** *represent* the kdtA and rfaQ transcripts was confirmed

If we consider the event denoted by the verb *represent*, **the phrase *the identified promoters* DOES NOT constitute a SOURCE variable for this event.**

Firstly, the verb *represent* corresponds more to a state rather than an *action* event; only the latter type of event can have a SOURCE.

Secondly, the phrase *from the identified promoters* is not actually a separate variable of the *represent* event at all; it merely provides additional information about the THEME of the event, i.e. *the two divergent transcripts.*

9.8 DESTINATION

This is the "companion" role to SOURCE. Variables assigned to this role have the following general characteristics:

- **The DESTINATION role corresponds phrases that specify to the *end point* of an event**
- **They are normally entities**
- **They are locations, but should not be confused with the LOCATION role,** which corresponds to where the *whole* event takes place.
- **They are typically preceded by the prepositions *to or into***
- **Unlike LOCATIONs, the preceding preposition SHOULD NOT be included in the annotated span of the variable**

Some examples are shown in examples (a) – (d).

(a) Transcription of gntT is activated by *binding* of the cyclic AMP (cAMP)-cAMP receptor protein (CRP) complex to [NP **a CRP binding site**]
(b) The *introduction* of a cysB allele, either on a plasmid or on an episome to [NP **the fusion strains**], resulted in the decrease of beta-galactosidase activity
(c) The repression is initiated by autophosphorylation of the sensor protein ArcB, followed by phosphoryl group *transfer* to [NP **the regulator ArcA**]
(d) P1 *transduction* of marA::Tn5 into [NP **a Mar mutant**] partially restored OmpF levels.

9.9 TEMPORAL

The TEMPORAL semantic role should be assigned to phrases with the following characteristics:

- **They situate the event in time**
- **They situate the event with respect to another event.**
- **They often begin with prepositions that indicate time or ordering of events, such as *during, following, before, after* or *at***
- **The preceding preposition (PP chunk) SHOULD BE INCLUDED within the annotated span of the variable,** as it is important to the interpretation of the phrase.

© University of Manchester and ILC-CNR

There are several types of temporal expression:

a) **Specification of the duration of an event**, as shown in (a).

(a) Analyses to quantitate the induction of this system show that derepression of the operon is first detectable 5 min after UV exposure, with the rate of synthesis *increasing* to four to six times the uninduced rate [PP **during**] [NP **the subsequent 30 min**].

2) **Situation of the event in time with respect to another event**. Some examples are shown in (b), (c) and (d).

(b) The Alp protease activity is *detected* in cells [PP **after**] [NP **introduction**] of plasmids carrying the alpA gene, which encodes an open reading frame of 70 amino acids.
(c) PhoB is known to be a transcriptional activator of the Pho regulon, expression of which is *activated* [PP **during**] [NP **phosphate starvation**].
(d) E . coli NM81 transformed with pJB22 had enhanced membrane Na+/H+ antiporter activity that was cold labile and that *decreased* very rapidly [PP **following**] [NP **isolation**] of everted vesicles.

3) **Specification that 2 or more things happen in parallel**, as illustrated in (e).

(e) Complementation of such a mutant with the cloned fragments *reversed* both phenotypes [PP **at**] [NP **the same time].**

If the temporal situation of an event with respect to another event also specifies a more precise timing, this timing should also be included within the annotated span. Two examples of this are shown in (f).

(f) Upon return to permissive temperature (30 degrees C), the transcripts *reappeared* coordinately [NP **about 15 min**] [PP **after**] [NP **the first synchronized initiation**] and then *declined* sharply again [NP **10 min**] [ADVP **later**].

Firstly, the *reappeared* event happens after the *initiation* event. There is also a specification of the amount of time that elapsed between these two events, i.e. 15 min. Hence, the complete phrase *about 15 min after the first synchronized initiation* is annotated as the TEMPORAL phrase associated with the *reappaeared* event. Secondly, the *declined* event occurs 10 minutes after the *reappeared* event, and so *10 min later* is marked as a TEMPORAL phrase associated with the *declined* event.

9.10 CONDITION

The CONDITION semantic role is appropriate for:

- **Phrases describing the environmental conditions which must hold in order for the event to take place**.

© University of Manchester and ILC-CNR

Environmental conditions can take a number of forms. **Three of the most common types are the following:**

1. **Changes in conditions that trigger the event.**
   - **Frequently preceded by the phrase *in response to,* but this should be excluded from annotated text span.** Examples are shown in (a) and (b)

   (a) Strains carrying a mutation in the crp structural gene fail to *repress* ODC and ADC activities in response to [NP **increased cAMP**] obtained by carbon source manipulation or cAMP supplementation of the growth medium

   (b) The Escherichia coli Ada protein *activates* sigma(70)-dependent transcription at three different promoters (ada, aidB, and alkA) in response to [NP **alkylation damage**] of DNA.

2. **Presence or absence of particular substances in the environment.**
   - **Frequently preceded by the phrases *in the presence of* or *in the absence of*, but these should be excluded from the text span that is marked to represent the variable.** Examples are shown in (c), (d) and (e).

   (c) The dcuB gene of Escherichia coli encodes an anaerobic C4-dicarboxylate transporter that is induced anaerobically by FNR, activated by the cyclic AMP receptor protein, and *repressed* in the presence of [NP **nitrate**] by NarL

   (d) A chromosomal deletion of gcvA resulted in the inability of cells to *activate* the expression of a gcvT-lacZ gene fusion when grown in the presence of [NP **glycine**] and an inability to *repress* gcvT-lacZ expression when grown in the presence of [NP **inosine**].

   (e) Here we show that OmpR, under certain conditions, could activate porin expression in the complete absence of [NP **EnvZ**].

3. **Characterisations of the conditions under which the event takes place. These may take the form of an adverb, but also often take the form of *Under x condtions*, where *x* characterizes the conditions in which the event takes place. In this case, *under* should be omitted from the annotated text span.** An example of a condition expressed by an adverb is shown in (f), whilst a condition in the form of *under x conditions* is shown in (g).

   (f) Expression of sdhCDAB (encoding succinate dehydrogenase) and lctD (encoding the flavin-linked L-lactate dehydrogenase) is *elevated* [ADVP **aerobically**] and *repressed* [ADVP **anaerobically**] in Escherichia coli

   (g) [PP Under] [NP **anaerobic conditions**] the narL gene product, in the presence of [NP **nitrate**], is known to *activate* transcription of the narC operon.

   In (g), there are actually 2 conditions specified: there is *nitrate* in addition to *anaerobic conditions.* In such case, the different conditions should be treated in the same way as lists. That is to say, both conditions should be assigned concept types and annotated as a single, discontinuous annotation marked with the CONDITION role. RATE

© University of Manchester and ILC-CNR

9.11 RATE

The RATE semantic role corresponds to phrases that have the following characteristics:

- **They describe changes in rates or levels that occur as part of the event.**
- **They normally have one of the following formats: *n-fold, n times* or *n %.***
- **In most cases, the change described by a RATE variable will apply to the THEME of the event.**
- **RATE variables are often preceded by prepositions, but this SHOULD NOT be included within the annotated span**
- **Rate *changes* are often preceded by the preposition *by*.** An example is shown in (a).

In (a), the rate change applies to the THEME of the *elevated* event.

(a) marR mutations that elevate marRAB transcription and engender multiple antibiotic resistance *elevated* inaA expression by [NP **10-**] [PP **to**] [ADVP **20-fold**] over that of the wild-type.

- **RATE variables may also correspond to the level *to which* one of the other variables has been increased or decreased during the event. In this case, the preposition *to* typically precedes the variable.** An example is shown in (b).

(b) Analyses to quantitate the induction of this system show that derepression of the operon is first detectable 5 min after UV exposure, with the rate of synthesis *increasing* to [ADVP **four to**] [NP **six times the uninduced rate**] during the subsequent 30 min

- **In other cases, RATE variable phrases can stand alone, without any preceding preposition.** This is illustrated in (c) and (d).

(c) Furthermore, in a delta envZ strain of E. coli, containing the envZ Val-243 plasmid, ompC expression is *elevated* [ADVP **7-fold**] relative to that found in cells carrying the wild-type envZ plasmid.

- **RATE variables can also apply to nominalised verbs. In these cases, the amount should *only* be identified as a separate variable-denoting phrase if a specific rate of change is specified**. If the change is less precise, e.g. if *10-fold* in (d) was replaced by *small,* then *small* should not be separately identified a variable-denoting phrase.

(d) Overexpression of the sfs1 gene in MK2001 resulted in a [NP **10-fold** *increase*] of amylomaltase.

IMPORTANT NOTE: Not all phrases of the forms *n-fold, n times* or *n %* should be marked as RATE variable, e.g. if they merely express a quantity of another variable. RATE variables normally only occur with verbs or nominalised verbs that imply some sort of *change* in rate or level e.g. "increase", "decrease" etc. In (f), although the emboldened

© University of Manchester and ILC-CNR

phrases express percentages, they DO NOT correspond to RATE variables. This is because they are expressing *quantities* of the THEMEs of the *expressed* events. They are *not* describing rate or level changes that occur as part of the *expressed* events.

(e) Mar mutants of an ompF-lacZ operon fusion strain *expressed* **50 to 75%** of the beta-galactosidase activity of the isogenic non-Mar parental strain, while Mar mutants of a protein fusion strain *expressed* **less than 10%** of the enzyme activity in the non-Mar strain.

However, it should be noted that percentages *can* act as RATE variables in other types of sentence. For example, in a sentence of the form *X increased Y by 10%*, the RATE of the *increased* event would be *10%.*

Care should also be taken that the RATE variable is associated with the *correct* event, if there is more than one event in the sentence. An example is shown in (g):

(f) Induction at 42 degrees C led to **100-fold** *overproduction* of EIImtl.

In (g), there are 2 events, one denoted by the verb *led* and the other denoted by the nominalised verb *overproduction.* The RATE variable *100-fold* belongs to the *overproduction* event, rather than the *led* event.

9.12 DESCRIPTIVE

Variables of the DESCRIPTIVE category can be best characterized as follows:

- **They describe *characteristics or behaviour* of one of the other variables in the event.**
- **Normally apply to either the AGENT or the THEME of the event. We thus distinguish two separate sub-roles, i.e. DESCRIPTIVE-AGENT and DESCRIPTIVE-THEME.**

There are 2 main contexts in which the DESCRIPTIVE role should be assigned

1) **Descriptions of characteristics or behaviour that normally follow the preposition *as*. Such descriptions can apply either to the AGENT or THEME of the event.** Examples are shown in (a) and (b).

In (a), the descriptive phrase (*a formate-dependent regulator*) refers to the AGENT of the verb *acts* (i.e. HyfR), hence the role assigned should be DESCRIPTIVE-AGENT.

(a) It is likely that HyfR *acts* as [NP **a formate-dependent regulator**] of the hyf operon

Another type of sentence where the use of the DESCRIPTIVE-AGENT role is appropriate is shown in b):

(b) Mucous cells *participate* in [NP **the interaction**] with enteropathogens

In (b), *Mucous cells* is the AGENT and *the interaction* is the DESCRIPTIVE-AGENT, as it is providing descriptive information about what the AGENT is doing.

© University of Manchester and ILC-CNR

In example (c), the phrase *a revertant* is describing a characteristic of *the recA gene*, which is the THEME of the *isolated* event. Therefore, the phrase *a revertant* is annotated as the DESCRPTIVE-THEME.

(c) A mutant strain of E. coli displaying altered regulation of the recA gene was *isolated* as [NP **a revertant**] of a lexA3 recA200 double mutant which showed improved DNA repair and recombination functions.

A further type of sentence where the behaviour of the THEME is being described is illustrated in (d).

(d) Uridine is *involved* in [NP **the recognition**] of tRNA substances.

Here, *uridine* is the THEME of *involved*: it is not *doing* the *involving,* but rather it *is involved.* The rest of the sentence described *what* the theme is involved in, i.e. the recognition of tRNA substances. This can be seen as information about behaviour, and hence it is appropriate to assign the role DESCRIPTIVE-THEME to the chunk *the recognition.*

2) **Descriptions in events that correspond to states, rather than actions**. Such events have the following characteristics:

- **There is no AGENT**
- **The subject of the verb corresponds to the THEME of the event**
- **The DESCRIPTIVE-THEME is assigned to variables that correspond to characteristics or attributes of the THEME.**
- **The DESCRIPTIVE-THEME variable is normally the object of the verb**

Examples of such sentences are shown in (e) and (f). In both cases, the emboldened phrase corresponds to the DESCRIPTIVE-THEME.

(e) In addition, the ompR-lacZ fusion *exhibits* [NP **a dominant OmpR- phenotype**].
(f) An Escherichia coli genomic library *composed* of [NP **large DNA fragments**] (10-15 kb) was constructed using the plasmid pBR322 as vector.

The *meaning* of certain verbs/nominalised verbs (such as those in (a) – (d) above) is such that the event itself is focused on providing a description of the AGENT or THEME. In this case the descriptive phrases are treated as actual variables of the event; the descriptive phrases are *required* if the event is to make sense (or at least the meaning of the event would be different if they are not present).

In other cases, the event itself is **NOT** focussed on providing descriptive information about the AGENT or THEME, but it is still possible to include extra descriptive information within the sentence, as shown in (e), (f) and (g). **NONE** of the **emboldened** phrases correspond to DESCRIPTIVE-AGENT or DESCRIPTIVE-THEME

(g) The global regulator CsrA, **an RNA binding protein**, *coordinates* central carbon metabolism.

© University of Manchester and ILC-CNR

The event described by the verb *coordinates* has an AGENT (*The global regulator CsrA*) and a THEME (*central carbon metabolism*). **There is additionally a descriptive phrase relating to the AGENT (i.e. *an RNA binding protein*). However, this phrase should NOT be annotated as DESCRIPTIVE-AGENT, as it does not constitute a separate piece of information about the *coordinates* event.** It merely provides extra information about the AGENT, and there is no difference in the meaning of the even if this descriptive phrase is omitted.

A further example is shown in (h)

(h) These promoters *generated* transcripts **with 5' ends separated by 289 bases**

Here, *These promoters* is the AGENT of *mediated*, whilst *transcripts* is the THEME. **The phrase** *with 5' ends separated by 289 bases* **provides descriptive information about the THEME, but does NOT contribute new information about the description of the event. Hence, it should NOT be annotated as the DESCRIPTIVE-THEME.**

Descriptive information in brackets also does NOT constitute a separate variable of the event, e.g.

(i) The FIS protein (**factor for inversion stimulation**) is known to *activate* the transcription of rRNA and tRNA operons in Escherichia coli

In (i), *The FIS protein* is the AGENT of *activate.* The information in brackets (factor for inversion stimulation) simply explains the FIS acronym, but does not constitute a separate event variable.

9.13 PURPOSE

The semantic role PURPOSE is appropriate to assign to variables that have the following characteristics:

- **Variables that specify *why* the event occurred, i.e. specifications of some sort of aim, purpose, goal or reason for the event occurring.**
- **The PURPOSE role always corresponds to an event of some kind, using either a verb, (see (a) and (c)), or a nominalised verb, (see (b)).**
- **Verbs that correspond to the PURPOSE role are normally in infinitive form (i.e. preceded by *to*)**
- **Nominalised verbs that correspond to purposes are often preceded by the preposition *for*.**

In (a), some unspecified (human) agent is using the fusion strains, and the *purpose* or *reason* for using them is to study the regulation of the cysB gene.

(a) The fusion strains were *used* [VP **to study**] the regulation of the cysB gene by assaying the fused lacZ gene product

In (b), the focus is the verb *required.* Some unspecified agent requires a chromosomal locus, and the reason for this requirement is to allow the alpA+ suppression to take place.

© University of Manchester and ILC-CNR

(b) We have used Tn10 and lambda placMu mutagenesis to identify a chromosomal locus, slpA, that is *required* for [NP **alpA+ suppression**] of delta lon.

Note that *suppression* is a nominalised verb and so should subsequently be annotated with its own variables.

In (c), the *purpose* of isolating the fragment was to complete the sequence of the cadA homolog.

(c) A 6.0-kb fragment overlapping the pJB22 insert was *isolated* [VP **to complete**] the sequence of the cadA homolog

Appendix A: Annotation Procedure

The main subtasks of the annotation process are as follows:

- Locating gene regulation events described by verbs and nominalised verbs
- Identifying variable-denoting phrases associated with each event
- Marking appropriate spans of text to represent these variables
- Assigning appropriate semantic roles to the variables
- Assigning categories to entities from the hierarchy

In order that the annotation of event variables is carried out as consistently and accurately as possible, it is recommended that a certain procedure or workflow for carrying out the annotation is adopted. This appendix provides a set of suggested steps to constitute this workflow.

1) **Start with the first sentence in the abstract**
   - **Is the sentence on the topic of gene regulation?**
     - **If yes, ensure that all parts of the sentence are fully understood.** If anything in the sentence is unclear, surrounding sentences should be read to help put the events described in the sentence into context. **Move to step 2).**
     - **If no, repeat step 1) with the next sentence in the abstract.**
2) **Locate the *main* verb of the sentence**. This is the verb that describes the main or most important event in the sentence. This may mean that verbs in the sentence are not annotated in sequential order. However, annotating the main event before annotating secondary events is easier and more intuitive

   REMEMBER: As long as the sentence is related to gene regulation, ALL verbs and nominalised verbs in the sentence should be annotated.
3) **Before beginning annotation, read through the *complete* sentence again, concentrating on the event denoted by the selected verb and trying to locate the phrases in the sentence that correspond to variables in the event.** This step is important to ensure that *all* variables of the event are located, and to ensure that no misinterpretations of variables occur. Consider sentence (a).

   (a) *Mutations affecting the BarA/UvrY two-component signal transduction system* ***decreased*** *csrB transcription*

   If the complete sentence is not read correctly when considering the *decreased* event, it would be easy to mistakenly mark *the BarA/UvrY two-component signal transduction*

© University of Manchester and ILC-CNR

system as the AGENT of the event, rather than the correct agent, i.e. *Mutations.* Section 7 of this document discusses how to identify variables in more complex sentences.

4) **Annotate each variable-denoting phrase of the event.** It is suggested that the AGENT and THEME of the event are annotated first, if they are present in the sentence, followed by phrases corresponding to other roles.
REMEMBER:
   - ***All* variables of the event *within the same sentence* should to be annotated. This includes:**
     a) Variables that do not correspond to one of the existing semantic roles (The UNDERSPECIFIED role should be assigned, together with a comment)
     b) Variables that don't correspond to a concept in the *concept hierarchy,* e.g. *we*
   - **Each variable should generally contribute a different type of information towards the description of the event. This means, for example that lists of items generally correspond to a SINGLE event variable.**

The annotation should proceed as follows:

i) **Mark an appropriate span of text to represent the variable**, according to the *Marking Phrases* guidelines in section 6.
REMEMBER:
   - **Spans should normally consists of complete chunks (single chunks wherever possible)**
   - **Short entity names are to be favoured over longer names or characterisations, if both are present within the sentence**
   - **Descriptive information about entities should not be included within the span**
   - **Where a variable consists of a list of entities, the span should consist of all items in the list, excluding commas and conjunctions etc.**
   - **LOCATION and TEMPORAL spans should include the preposition that precedes them, e.g. *in, after* etc.**

ii) **Assign an appropriate semantic role to the marked phrase**, or UNDERSPECIFIED if none of the roles in the current set seems appropriate. In the case, it is important to include a comment that explains the perceived function of the phrase in the event.
   - Section 7 provides a detailed description of the various roles, which should be read carefully before beginning annotation. **However, it is suggested that Appendix 2 of this document, "Quick Role Guide" is used as an aid when carrying out semantic role assignment.** It provides a tabular, quick reference guide to the semantic roles with useful reminders about typical phrase features, clues in the surrounding text etc.

5) **Re-examine each of the variable phrases marked during step 4). Further action is required if the variable corresponds either to an entity or an event, as follows:**
   - If the variable corresponds to an entity that is a biological concept, an appropriate category should be assigned from concept hierarchy (see section 4)

© University of Manchester and ILC-CNR

- If the variable corresponds to another, embedded event (denoted by a verb or nominalised verb), annotation of the variables of this event should be carried out by returning to step 3), but this time considering the embedded event.

REMEMBER:

- **There may be more than one level of event "embedding"; in this case, the variables of events at all levels of embedding should be annotated.** Consider sentence (b):

(b) *It was observed that an increase in the copy number of the uxuR gene* **results** *in an increased repression of beta-galactosidase synthesis*

In this sentence, the THEME of results is *an increased repression.* As *repression* is a nominalised verb, its own variables should be identified. The THEME of *repression* is another event, i.e. *synthesis,* whose own THEME is *beta-galactosidase.*

6) **Locate the next verb or nominalised verb to be annotated, and return to step 3)** If the verb just annotated was the main verb in the sentence, it should be verified whether there are any verbs/nominalised verbs to annotate which occur *before* the main verb in the sentence, before moving on to look at verbs after the main verb. The annotation process ends when all sentences in the abstract have been read.

© University of Manchester and ILC-CNR

Appendix B : Quick Semantic Reference Role Guide

Role Name	Description	Phrase Type(s)	Clues
**AGENT**	Reponsible for event;<br>Only assigned when event denotes action	Entity or event	Typically subject of verb,<br>Follows *by* in passive sentences
1) **The narL gene product** *activates* the nitrate reductase operon<br>2) This operon is negatively *controlled* by **the uxuR regulatory gene product.**<br>3) The control of uvrB was found to *result* from **direct repression** by the lexA gene product<br>4) *Phosphorylation* of OmpR by **the osmosensor EnvZ** modulates …			
**THEME**	Directly involved in event but not responsible for it. Either:<br>1)Affected by or results from "action" event; ***or***<br>2)Focus of descriptions of states	Entity or event	Object of verb in "action" events,<br>subject in descriptions of states,<br>subject in passive sentences
1) The narL gene product *activates* **the nitrate reductase operon**<br>2) **recA protein** was *induced* by UV radiation<br>3) The release of 4.5 S RNA from polysomes is affected by antibiotics that *inhibit* **protein *synthesis***<br>4) **The recA430 protein** *possesses* ssDNA-dependent rATP activity			
**MANNER**	***Method or way*** in which event is carried out, normally biological or experimental process.<br>**Don't confuse with INSTRUMENT**	Event (process),<br>adverb,<br>direction,<br>*in vitro, in vivo* etc.	Events typically follow *by, through, via* or *using*
1) Using **random Tn10 insertion mutagenesis**, we *isolated* an Escherichia coli mutant strain affected in the regulation of lysU<br>2) CsrA *stimulates* UvrY-dependent activation of csrB expression by **BarA-dependent and -independent mechanisms**.<br>3) These results suggest that transcription of the fadL gene is **osmotically** *regulated* by the OmpR-EnvZ two-component system<br>4) The gene is *transcribed* **counterclockwise** on the standard Escherichia coli map<br>5) These results lead us to conclude that EnvZ and OmpR *act* in **sequential fashion**			
**INSTRUMENT**	***Entity* used by agent** to carry out event.<br>**Don't confuse with MANNER**	Entity	Typically follows *with, with the aid of, via, by, through, using*
1) EnvZ *functions* through **OmpR** to control porin gene expression in Escherichia coli K-12<br>2) We have *isolated* a strain … with the aid of **the Casadaban Mud phage** .			
**LOCATION**	Where the ***complete*** event takes place.<br>**Don't confuse with SOURCE/DESTINATION**	Entity	Typically begins with *in, at, on, near* or *between*
1) Phosphorylation of OmpR by the osmosensor EnvZ *modulates* expression of the ompF and ompC genes **in Escherichia coli**<br>2) The fic gene was *located* **near rpsL** on the E. coli K-12 map<br>3) The mutant (alc-24) was *located* **between srl and recA200**			

© University of Manchester and ILC-CNR

**SOURCE**	Where the event ***starts***<br>**Don't confuse with LOCATION**	Entity	Typically follows *from*
1) To determine the expression of BMI1, a BMI1-LacZ construct was *extracted* from **pBR322 plasmid** and inserted into E.coli chromosomal DNA.<br>2) *Transduction* of the marA region from **a Mar strain** ...<br>3) The transcriptional direction of the uxuR gene was *deduced* from **the restriction pattern**			
**DESTINATION**	Where the event ***ends***<br>**Don't confuse with LOCATION**	Entity	Typically follows *to* or *into*
1) Transcription of gntT is activated by *binding* of the cyclic AMP (cAMP)-cAMP receptor protein (CRP) complex to **a CRP binding site**<br>2) To determine the expression of BMI1, a BMI1-LacZ construct was extracted from pBR322 plasmid and *inserted* into **E.coli chromosomal DNA**.			

**TEMPORAL**	Situates event in time possibly with respect to another event	Normally an event or time interval	Often preceded by *during, before* or *after*
1) The Alp protease activity is *detected* in cells **after introduction** of plasmids carrying the alpA gene<br>2) The rate of synthesis *increased* to four to six times the uninduced rate **during the subsequent 30 minutes**<br>3) Complementation of such a mutant with the cloned fragments *reversed* both phenotypes **at the same time**			
**CONDITION**	Conditions or changes in conditions under which the event takes place; presence or absence of substances in environment	Entity (e.g. substance present in environment), event (e.g. change in conditions) or adverb	Conditions often in the form of *under x conditions* or adverb Substances typically follow *in the presence/absence of.* Changes in conditions typically follow *in response to.*
1) Strains carrying a mutation in the crp structural gene fail to *repress* ODC and ADC activities in response to **increased cAMP**<br>2) The dcuB gene of E. Coli encodes an anaerobic C4-dicarboxylate transporter that is *repressed* in the presence of **nitrate** by NarL<br>3) Under **anaerobic conditions**, the narL gene product is known to *activate* transcription of the narC operon			
**RATE**	Change in rate or level occurring as part of event. Normally applies to the THEME.	Typically of the form *n-fold*, *n times* or *n %*	May follow *by*
1) marR mutations that elevate marRAB transcription and engender multiple antibiotic resistance *elevated* inaA expression by **10- to 20-fold** over that of the wild-type.<br>2) The rate of synthesis *increases* to **four to six times the uninduced rate** during the subsequent 30 minutes<br>3) ompC expression is *elevated* **7-fold**			

© University of Manchester and ILC-CNR

**DESCRIPTIVE-AGENT**	Describe characteristics or behaviour of AGENT of event	Entity or Event	Often follows *as*; object of verb in descriptions of states
1) It is likely that HyfR *acts* as **a formate-dependent regulator** of the hyf operon<br>2) Mucous cells *participate* in **the interaction** with enteropathogen.			
**DESCRIPTIVE-THEME**	Describe characteristics or behaviour of THEME of event	Entity or Event	Often follows *as;* Object in descriptions of states
1) A mutant strain of E.Coli *was isolated* as **a revertant** of lexA3 recA200 double mutant<br>2) Uridine is *involved* in **the recognition** of tRNA substances<br>3) The ompR-lacZ fusion *exhibits* **a dominant OmpR-phenotype**			
**PURPOSE**	Specifies *why* the event occurs, i.e. an aim, purpose, goal or reason for the event occuring	Event	Typically a verb in infinitive form or a nominalised verb following *for.*
1) The fusion strains were *used* **to study** the regulation of the cysB gene by assaying the fused lacZ gene product<br>2) We have used Tn10 and lambda placMu mutagenesis to identify a chromosomal locus, slpA, that is *required* for **alpA+ suppression** of delta lon.			

© University of Manchester and ILC-CNR
