# Supplementary material for: Construction of an annotated corpus to support biomedical information extraction
Source: BMC Bioinformatics. 2009 Oct 23;10:349. doi: 10.1186/1471-2105-10-349 (PMC2774701; doi:10.1186/1471-2105-10-349)
Supplement: Additional file 1 — GREC mini-website. This website provides brief details of the GREC and descriptions of the available corpus formats. It also provides links to download both the corpus and the annotation guidelines. [file 1471-2105-10-349-S1.ZIP › index.html]

xml version="1.0" encoding="iso-8859-1" ?


The GREC corpus


The GREC Corpus

# The GREC Corpus

## Download

The corpus in available for download in 2 formats:

- A standoff format, based on the BioNLP'09 Shared Task format- An XML format, based on the GENIA event annotation format

The annotation guidelines are also available to download.

## Background

Information Extraction (IE) is a component of text mining that facilitates knowledge discovery by automatically locating instances of interesting biomedical events from huge document collections. Effective IE systems require training data or annotated corpora, in which instances of biomedical events are explicitly identified in texts. The trained IE systems can then recognise instances of new events in texts, facilitating a number of text mining applications, such as pathway maintenance and semantic searching.

## The Corpus

The GREC corpus is a semantically annotated corpus of MEDLINE abstracts which is intended for training IE systems and/or resources which are used to extract *events* from biomedical literature.

The corpus has been manually annotated with events relating to *gene regulation* by biologists. Each event is centred on either a verb (e.g. *transcribe*) or nominalised verb (e.g. *transcription*) and annotation consists of identifying, as exhaustively as possible, the structually-related arguments of the verb or nominalised verb within the same sentence. Each event argument is then assigned the following information:

- A *semantic role* from a fixed set of 13 roles which are tailored to the biomedical domain.
- A biomedical concept type (where appropriate).

As a simple example, consider the following sentence:   
  
*The narL gene product **activates** the nitrate reductase operon*  
  
The sentence contains a single event, centred on the verb *activates*, with 2 arguments, i.e.:

1. *The narL gene product*
2. *the nitrate reductase operon*

The argument *The narL gene product* is assigned the semantic role *AGENT* and the biological concept *Protein*, whilst the argument *the nitrate reductase operon* is assigned the semantic role *THEME* and the biological concept *Operon*.

Other types of argument include:

- LOCATION, e.g. *In Escherichia Coli*, glnAP2 may be **activated** by NifA
- MANNER, e.g. *cpxA gene **increases** the levels of csgA transcription by dephosphorylation of CpxR*
- CONDITION, e.g. *Strains carrying a mutation in the crp structural gene fail to **repress** ODC and ADC activities in response to increased cAMP*

Full details of the annotation scheme can be found in the annotation guidelines.

## Contact

For any queries relating to the corpus, please contact:  
paul.thompson at manchester.ac.uk
