# Supplementary material for: Construction of an annotated corpus to support biomedical information extraction
Source: BMC Bioinformatics. 2009 Oct 23;10:349. doi: 10.1186/1471-2105-10-349 (PMC2774701; doi:10.1186/1471-2105-10-349)
Supplement: Additional file 1 — GREC mini-website. This website provides brief details of the GREC and descriptions of the available corpus formats. It also provides links to download both the corpus and the annotation guidelines. [file 1471-2105-10-349-S1.ZIP › xml.html]

xml version="1.0" encoding="iso-8859-1" ?


The GREC corpus- XML Annotation


The GREC Corpus

# GREC Corpus - XML annotation format

## Download

The XML format of the annotation may be downloaded here: GREC\_XML.zip

The use of the corpus is subject to NaCTeM's Terms and Conditions, and in particular Section 8, regarding the use of NLM databases.

The directory contains 3 subdirectories:

- **Ecoli** - Contains abstracts on the subject of Ecoli.
- **Human** - Contains abstracts on the subject of Human.
- **GRECResources** - Contains the DTD of the XML annotation files (GREC\_event.dtd)

## Corpus description

The XML annotation format for the corpus is based on the GENIA event annotation format with some minor modifications.

Two levels of annotation of the target text are expressed within each file, i.e.

- text-bound event arguments and other annotated biological concepts
- event annotations

An example of an annotated sentence within the XML file is shown below:

```
...
<sentence id="S7">
   <term sem="SPAN" id="T10" lex="The_loss">The loss</term> 
   of TreR function led to derepression of 
   <term sem="Gene" id="T11" lex="treB">treB</term> 
   encoding 
   <term sem="SPAN" id="T12" lex="an_enzymeIITre">an enzymeIITre</term> 
   of the PTS for trehalose and of 
   <term sem="Gene" id="T13" lex="treC">treC</term> 
   encoding 
   <term sem="Enzyme" id="T14" lex="TreC">TreC</term>
   , the cytoplasmic trehalose-6-phosphate hydrolase.
</sentence>
<event id="E6">
  <type class="GRE" />
  <Agent idref="T10" />
  <Theme idref="E7" />
  <clue>The loss of TreR function <clueType>led</clueType> 
  to derepression of treB encoding an enzymeIITre of the PTS for trehalose and 
  of treC encoding TreC, the cytoplasmic trehalose-6-phosphate hydrolase.</clue>
</event>
<event id="E7">
  <type class="Gene_Activation" />
  <Theme idref="T11" idref1="T13" />
  <clue>The loss of TreR function led to <clueType>derepression</clueType> 
  of treB encoding an enzymeIITre of the PTS for trehalose and of treC encoding TreC, the 
  cytoplasmic trehalose-6-phosphate hydrolase.</clue>
</event>
...
```

Each sentence of the abstract is contained within a *<sentence>* element. Biological concepts and other event arguments are annotated inline, indicated by *<term>* elements. Event arguments may or may not correspond to biological concepts. In other cases, a biological concept may form only part of an event argument. Elements of type *<term>* correspond to all annotated biological concepts, together with other text spans that consistute event arguments.

Each *<term>* element has the following attributes:

- **sem** - The biological concept type assigned to the span, or "SPAN" if no concept type has been assigned.
- **id** - A unique id for the span, beginning with "T"
- **lex** - The value of the text span, with spaces replaced with underscores

Following the *<sentence>* element, the events in the sentence are listed, each within an *<event>* element.
Each event has a unique id, starting with an "E". Within the *<event>* element, there are the following elements:

- **Type**- The type assigned to the event is indicated by the *class* attribute. The general type "GRE" (Gene Regulation Event) is generally assigned to verb trigger words, as "top level" events centred on verbs are not assigned types. Embedded events (those which form an argument to another event, normally nominalised verbs) may be assigned a concept type. In this case, the type is indicated (as in event E7 above)
- **Semantic Arguments** - There is an element corresponding to each annotated argument of the event, which is named according to the semantic role assigned to the argument, e.g. *Agent*, *Theme*, *Location*, *Condition* etc. Each element has one or more attributes, whose values correspond the id(s) of the argument which fills the role. The attributes are named *idref*, *idref1*, *idref2* etc. The value ot each attibute begins either with a "T", indicating that the argument span corresponds to one of the *<term>* elements, or it may begin with an "E", indicating that the argument is an embedded event whose structure is described in another *<event>* element.
- **Clue** - This element contains the complete sentence containing the event. The *<clueType>* element surrounds the verb/nominalised verb on which the event is cented.

## Discontinuous spans

The *idref* attribute is always present, whilst *idref1*, *idref2* etc. are only present if the event argument corresponds to two or more *discontinuous* spans of text. This is the case, for example, when an argument consists of a list of items, the annotator is required to annotate discontinuous spans, consisting of the items in the list, minus any conjunctions or punctuation. In event E7 above, the THEME of *derepression* consists of the two spans *treB* and *treC*, which are assigned the ids of T11 annd T13 respectively. In the *Theme* element of the event, the value of the attribute *idref* is this *T11*, whilst the value of *idref1* is *T13*.
