# Supplementary figures and images for: Construction of an annotated corpus to support biomedical information extraction
Source: BMC Bioinformatics. 2009 Oct 23;10:349. doi: 10.1186/1471-2105-10-349 (PMC2774701; doi:10.1186/1471-2105-10-349)

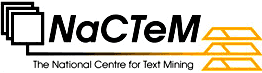

Supplement: Additional file 1 — GREC mini-website. This website provides brief details of the GREC and descriptions of the available corpus formats. It also provides links to download both the corpus and the annotation guidelines. [file 1471-2105-10-349-S1.ZIP › images/nactemlogo.gif]
