# Supplementary material for: Construction of an annotated corpus to support biomedical information extraction
Source: BMC Bioinformatics. 2009 Oct 23;10:349. doi: 10.1186/1471-2105-10-349 (PMC2774701; doi:10.1186/1471-2105-10-349)
Supplement: Additional file 1 — GREC mini-website. This website provides brief details of the GREC and descriptions of the available corpus formats. It also provides links to download both the corpus and the annotation guidelines. [file 1471-2105-10-349-S1.ZIP › GREC_Standoff.zip!/GREC_Standoff/README.html]

GREC Corpus - Standoff annotation format


# GREC Corpus - Standoff annotation format

The use of the GREC corpus is subject to NaCTeM's Terms and Conditions, and in particular Section 8, regarding the use of NLM databases.

The directory contains two sub-directories, named *Ecoli* and *Human*, which contain the abstracts on the subjects of E.coli and *Human*, respectively. For each abstact, there are 3 files, named according to the PMID of the abstract, with the following suffixes:

- **.txt** - Contains the original text of the abstract, with 2 lines. The first line contains the abstract title, whilst the second line contains the body of the abstract.
- **.a1** - Contains the named entity and event argument text spans (see below for more information)
- **.a2** - Contains annotations relating to events (see below for more information>

The standoff annotation format for the corpus is based on the BioNLP'09 Shared Task format with some minor modifications.

Two levels of annotation of the target text are expressed using stand-off style annotations, stored separately from the target text files. In the stand-off annotation files, each annotation is specified on a separate line. The types of annotations are as follows:

- text-bound event trigger annotions (i.e. verbs and nominalised verbs), event arguments and other annotated biological concepts (IDs beginning with "T").
- event annotations (IDs beginning with "E").

For each annotated abstract, 2 standoff annotation files are produced, with the suffixes "a1" and "a2".

## a1 Files

The "a1" files contain the event arguments and annotated biological concepts. An example is shown below:   
  

```
T1	Activator 0 4		EnvZ
T2	SPAN 15 27		through OmpR
T3	Regulator 23 27		OmpR
T4	Gene 39 49		porin gene
T5	SPAN 61 85		in Escherichia coli K-12
T6	Wild_Type_Bacteria 64 85	Escherichia coli K-12
T7	Regulator 88 125	The regulatory proteins OmpR and EnvZ
T8	Gene 170 179		the genes
T9	Activator 284 288	OmpR
```

Each line begins with a unique ID for the annotation. This is followed by a TAB character and then a SPACE-separated triple, consisting of the annotation type, its start offset and end offset. Another TAB is followed by the text span corresponding to the annotation.

Event arguments may or may not correspond to biological concepts. In other cases, a biological concept may form only part of an event argument. In the "a1" files, both annotated biological concepts and other spans which constitute event arguments are listed. Spans which do not correspond to biological concepts are assigned the type SPAN. In the example above, annotation T5 , i.e. *in Escherichia coli K-12* corresponds to an event argument. It is not a biological concept itself, but contains the biological concept *Escherichia coli K-12*, which is listed as a separate annotation (T6) with its concept type specified.

## a2 Files

The "a2" files contain annotations relating to events. An example is shown below:

```
T13	Gene_Activation 263 273	activation
T14	GRE 296 304	requires
T15	GRE 309 317	function
T16	Regulation 540 549	regulated
T17	GRE 815 824	exhibited
T18	Gene_Activation 849 859	activation
T19	GRE 1062 1070	effected
T20	GRE 1079 1086	control
E1	Gene_Activation:T13 Theme:T1
E2	GRE:T14 Agent:E1 Theme:E3
E3	GRE:T15 Theme:T2
E4	Regulation:T16 Agent:T4 Theme:T3
E5	GRE:T17 Agent:T6 Theme:E6 Manner:T7
E6	Gene_Activation:T18 Theme:T8 Location:T9
E7	GRE:T19 Theme:T10 Descriptive-Theme:E8
E8	GRE:T20 Theme:T11,T12
```

At the top of the file are annotations corresponding to event trigger words. These take the same format as the annotations in the "a1" files, including IDs beginning with "T". The general type "GRE" (Gene Regulation Event) is generally assigned to verb trigger words, as "top level" events centred on verbs are not assigned types. Embedded events (those which form an argument to another event, normally nominalised verbs) may be assigned a concept type. In this case, the type is indicated (as in E1, E4 and E6 above).

At the bottom of the files are the event annotations, denoted with IDs beginning with "E". The format of these lines is a frame-like format. Following the ID and the TAB character, there is a SPACE-separated n-tuple. The first part of this n-tuple consists of the event type, separated by a colon from the ID of the event trigger word. The remaining parts of the n-tuple correspond to the arguments of the event, consisting of the semantic role assigned to argument, separated by a colon from the ID(s) of the arguments. These IDs may begin with "T", referring to annotations in the "a1" file, or they may begin with "E", indicating an embedded event, which is also listed within the "a2" file.

## Discontinuous spans

It is possible for event arguments to have more than one ID (as in the THEME of event E8), corresponding to 2 or more discontinuous spans ot text. For example, when an argument consists of a list of items, the annotator is required to annotate discontinuous spans, consisting of the items in the list, minus any conjunctions or punctuation. For example,
